# Novel M23 peptidases Pgp4, Pgp5, and Pgp6 contribute to helical cell shape in *Campylobacter jejuni*

**DOI:** 10.3389/fmicb.2025.1641976

**Published:** 2025-09-09

**Authors:** Chang Sheng-Huei Lin, Jenny Vermeulen, Jacob Biboy, Erin C. Gaynor, Waldemar Vollmer, Emilisa Frirdich

**Affiliations:** ^1^Department of Microbiology and Immunology, University of British Columbia, Vancouver, BC, Canada; ^2^Centre for Bacterial Cell Biology, Biosciences Institute, Newcastle University, Newcastle upon Tyne, United Kingdom; ^3^Institute for Molecular Bioscience, The University of Queensland, Brisbane, QLD, Australia

**Keywords:** *Campylobacter jejuni*, peptidoglycan, M23 peptidase, bacterial morphology, DD-endopeptidase, DD-carboxypeptidase, pathogenic attributes

## Abstract

The helical morphology of *Campylobacter jejuni* is maintained by its peptidoglycan (PG) layer and influences its success as a pathogen. Periplasmic PG hydrolases that cleave the PG glycan backbone and peptide sidechains (such as carboxypeptidases and endopeptidases) are critical for proper cell function and/or growth and are important in the PG remodeling required for cell shape generation and any morphological alterations. The *C. jejuni* shape is determined by PG hydrolases Pgp1 (DL-carboxypeptidase), Pgp2 (LD-carboxypeptidase) and Pgp3 (DD-carboxypeptidase/DD-endopeptidase), as well as a group of M23 peptidase domain containing proteins with previously uncharacterized activity: CJJ81176_1105, CJJ81176_1228, and CJJ81176_0166. Using a PG cleavage assay, we showed that 1105 and 1228 have DD-carboxypeptidase/DD-endopeptidase activity, and 0166 is a DD-carboxypeptidase. We renamed 1105, 1228, and 0166 to Pgp4 (peptidoglycan peptidase 4), Pgp5, and Pgp6, respectively. Pgp6 is the first described *C. jejuni* M23 peptidase with substrate selectivity on monomeric pentapeptides. Sequence comparisons between the DD-carboxypeptidase Pgp6 and the DD-carboxypeptidase/DD-endopeptidase Pgp3 (with an available crystal structure) and their corresponding orthologs revealed that Pgp6 contains insertion sequences in the M23 peptidase domain not present in Pgp3. Modeling of Pgp6 predicted that the insertion sequences would restrict the active site groove, only allowing entrance of a smaller substrate. This provides a possible explanation for the lack of Pgp6 DD-endopeptidase activity. To our knowledge, Pgp6 is the first reported DD-carboxypeptidase in the M23 peptidase superfamily. Deletions in *pgp4, pgp5*, and *pgp6* resulted in mutants with varying curved rod morphologies and changes in PG muropeptide profiles in comparison to wild type and each other. Using these mutants, we examined the effect of deleting these genes on *C. jejuni* properties affecting pathogenesis and survival: motility, biofilm formation, autoagglutination, the ability to transition to a coccoid form, growth under varying pH, susceptibility to antimicrobial compounds, and adherence, invasion and intracellular survival in human epithelial cells. Each mutant showed distinct phenotypic changes to each other, indicating they are not functionally redundant. This also further supports the correlation between *C. jejuni* morphology and morphology-related genes with pathogenic potential.

## 1 Introduction

*Campylobacter jejuni* is the leading bacterial cause of gastroenteritis worldwide. *C. jejuni* is a highly motile Gram-negative bacterium belonging to the *ε*-Proteobacteria. It has a characteristic helical morphology in its most pathogenic form, but can also adopt a filamented helical rod morphology (Justice et al., 2008; Apel et al., 2012; Cameron et al., 2012; Frirdich and Gaynor, 2013; Ghaffar et al., 2015; Khan et al., 2022) or coccoid form under stress (Svensson et al., 2008; Jackson et al., 2009; Ikeda and Karlyshev, 2012; Frirdich and Gaynor, 2013; Frirdich et al., 2019). Changes to the helical shape affect *C. jejuni* physiology and pathogenic attributes, thereby affecting its success in causing disease (discussed below) (Cameron et al., 2012; Frirdich et al., 2012; Frirdich et al., 2014; Ha et al., 2016; Stahl et al., 2016; Frirdich et al., 2019).

Peptidoglycan (PG) is a rigid, but flexible mesh-like sacculus that surrounds the bacterial cytoplasmic membrane (Vollmer et al., 2008a). It provides the mechanical strength to withstand turgor pressure and is also responsible for maintaining cell shape. The basic PG structure is conserved in bacteria and is composed of glycan strands of β1,4-linked N-acetylglucosamine (GlcNAc) and N-acetylmuramic acid (MurNAc) residues cross-linked by short peptides. In Gram-negative bacteria, glycan strands terminate with 1,6-anhydro MurNAc (MurNAcAnh) residues and the peptides have the sequence L-Ala-D-iso-glutamate-meso-diaminopimelic acid (mDAP)-D-Ala-D-Ala and are attached to the MurNAc residue (Vollmer et al., 2008a). The terminal D-Ala residue is cleaved off during the transpeptidation reaction that results in the cross-linking of adjacent glycan strands. In *C. jejuni*, cross-links occur between an mDAP_3_ of one stem peptide with a D-Ala_4_ of another (DD-cross-links) with no LD cross-links being detected (Frirdich et al., 2012).

Periplasmic PG hydrolases that cleave the PG glycan backbone (glycosidases) and peptides [carboxypeptidases (CPases), endopeptidases (EPases) and amidases] are critical for proper cell function and/or growth and are important in the PG remodeling required for cell shape generation and any alterations to the cell shape such as during cell division (Uehara and Bernhardt, 2011; Typas et al., 2012; Frirdich and Gaynor, 2013; Do et al., 2020; Egan et al., 2020). PG remodeling can affect a bacterium’s ability to interact with the environment and with its host, thereby influencing its survival and virulence capabilities (Wyckoff et al., 2012; Frirdich and Gaynor, 2013; Wheeler et al., 2014; Juan et al., 2018; Do et al., 2020). CPases cleave C-terminal amino acids, EPases hydrolyze the amide bonds between two amino acids, and amidases cleave the bond between the glycan backbone and the peptide stem (Vollmer et al., 2008b). CPases and EPases are further classified by the chirality of the peptide bond hydrolyzed (DD-, DL-, or LD-) (Vollmer et al., 2008b). During the remodeling process, the pentapeptide can be trimmed to tetra-, the tetra- to tri- and the tri- to the dipeptide by DD-CPases, LD-CPases, and DL-CPases, respectively. The 4-3 cross-link is cleaved by a DD-EPase. The structures and abundance of PG peptides generated by a particular complement of PG hydrolases for any given wild type bacterial species gives rise to the characteristic profile of the disaccharide peptides (muropeptides) released by a muramidase during analysis (Vollmer et al., 2008a).

Seven PG hydrolases have been identified in *C. jejuni*, including six peptidases: Pgp1 (Frirdich et al., 2012), Pgp2 (Frirdich et al., 2014), Pgp3 (Min et al., 2020), Pgp4 (CJJ81176_1,105) (Esson et al., 2017; Frirdich et al., 2017; Frirdich et al., 2023), Pgp5 (CJJ81176_1,228) (Stahl et al., 2016; Frirdich et al., 2023), Pgp6 (CJJ81176_0166) (Frirdich et al., 2023) and one amidase AmiA (Frirdich et al., 2019). With the determination of the biochemical activity of 1105, 1228 and 0166 in this study, these enzymes were renamed Pgp4 (peptidoglycan peptidase 4), Pgp5 and Pgp6, respectively, and are referred to as such from now on. The *C. jejuni* helical shape is generated by the PG hydrolases Pgp1 and Pgp2 (Frirdich et al., 2012; Frirdich et al., 2014). Deletion mutants in *pgp1* and *pgp2* have a rod-shaped morphology. Pgp1 is a DL-CPase cleaving tri- to dipeptides and Pgp2 is an LD-CPase cleaving tetra- to tripeptides. The degree of curvature of the *C. jejuni* cell is dictated by PG hydrolases belonging to the M23 peptidase family: these include Pgp3 (Min et al., 2020), Pgp4 (Esson et al., 2017; Frirdich et al., 2017; Frirdich et al., 2023), Pgp5 (Stahl et al., 2016; Frirdich et al., 2023) and Pgp6 (Frirdich et al., 2023). Pgp3 was demonstrated to have DD-CPase and DD-EPase activities (Min et al., 2020). The gene deletion mutant strains in ∆*pgp3*, ∆*pgp4*, ∆*pgp5*, and ∆*pgp6*, displayed various changes in morphology resulting in altered curved rod morphologies, but not rod-shaped morphologies like ∆*pgp1* and ∆*pgp2*. Unlike with Pgp1 and Pgp2, specific assignments of hydrolase function of Pgp4, Pgp5 and Pgp6 from the muropeptide profile of deletion mutants were not possible (Frirdich et al., 2023).

Traditionally, the helical shape of *C. jejuni* was postulated as being important for burrowing through intestinal mucus, but the role of *C. jejuni* morphology in pathogenesis could only begin to be examined experimentally with the discovery of the rod-shaped ∆*pgp1* and ∆*pgp2* mutants (Frirdich et al., 2012; Frirdich et al., 2014). Both mutants showed changes in attributes affecting *C. jejuni* transmission (motility, biofilm formation), coccoid formation in the case of ∆*pgp1*, chick colonization and host interactions (Frirdich et al., 2012; Frirdich et al., 2014). They showed differential activation of cytoplasmic human nucleotide-binding oligomerization domain (Nod) receptors that recognize PG molecules as well as increased secretion of the proinflammatory chemokine IL-8 from epithelial cell infections by ∆*pgp1* (Frirdich et al., 2012; Frirdich et al., 2014). Despite having a similar rod-shaped morphology, Pgp1 and Pgp2 have distinctive hydrolase activities and the mutants have different PG muropeptide profiles (Frirdich et al., 2012; Frirdich et al., 2014). This explains some of their differing pathogenic attributes. Neither ∆*pgp1* nor ∆*pgp2* mutants were defective in adherence, invasion or intracellular survival within cultured intestinal epithelial cells (Frirdich et al., 2012; Frirdich et al., 2014). Despite this, both ∆*pgp1* and ∆*pgp2* mutants were non-pathogenic in a *Sigirr*−/− (Single IgG IL-1 Related Receptor defective) mouse model of *C. jejuni* infection (Stahl et al., 2014; Stahl et al., 2017). The rod-shaped mutants while able to colonize like wild type were unable to penetrate intestinal crypts or induce inflammation and disease pathology (Stahl et al., 2017). Interestingly, the curved rod mutant (∆*pgp5*) behaved like wild type (Stahl et al., 2017). Beyond this experiment, the effects of changes on the degree of helical curvature (as opposed to the complete loss of curvature as in rod-shaped mutants) on *C. jejuni* biology have not been examined.

Our previous work described the identification of Pgp4, Pgp5 and Pgp6 as potential PG hydrolases, as well as the morphological and muropeptide changes associated with *pgp4*, *pgp5* and *pgp6* deletion and overexpression (Frirdich et al., 2023) (morphological changes are shown in Supplementary Figure 1). This study expands on this by establishing the biochemical activity of Pgp4, Pgp5, and Pgp6 and examining how these enzymes influence *C. jejuni* pathogenesis. Using a PG cleavage assay, we demonstrated that Pgp4 and Pgp5, like Pgp3 (Min et al., 2020), have DD-CPase and DD-EPase activities, while Pgp6 is the first identified M23 peptidase with only DD-CPase activity (Razew et al., 2022). Sequence analysis and protein modeling suggest that Pgp6 contains four insertion sequences (SEQ1-4) not present in the M23 domain of the Pgp3, Pgp4 and Pgp5 M23 peptidases with both DD-CPase and DD-EPase activities. A Pgp6 AlphaFold model suggests that the insertion sequences may be involved in decreasing the size of the active site groove which may restrict substrate selectivity to the smaller monomeric pentapeptide.

Alterations in the levels of Pgp4, Pgp5 and Pgp6, as well as the resultant changes in cell morphology and PG muropeptide profile (Frirdich et al., 2023) had unique effects on the biological and pathogenic properties of *C. jejuni*. The properties examined included motility, biofilm formation, autoagglutination, cell surface hydrophobicity, calcofluor white (CFW) reactivity, pH survival, resistance to antimicrobial compounds, ability to transition to the coccoid form, and adhesion, invasion and intracellular survival in an epithelial cell line. Together, the phenotypic analyses of the mutant strains highlight that changes to the degree of curvature of the *C. jejuni* helical cell shape and not only the helical shape itself modulate *C. jejuni* physiology and pathogenesis.

## 2 Materials and methods

### 2.1 Bacterial strains and growth conditions

The bacterial strains and plasmids used in this study are described in Supplementary Table 1. Briefly, mutants were constructed by replacing part of the gene with a non-polar kanamycin (Km) resistance cassette (*aphA-3*) (Stahl et al., 2016; Frirdich et al., 2017; Frirdich et al., 2023). Complemented and overexpression strains were generated by expressing the gene at the rRNA spacer locus of the mutant or wild type strain, respectively (Stahl et al., 2016; Frirdich et al., 2017; Frirdich et al., 2023). For complementation/overexpression, the *pgp4*, *pgp5*, and *pgp6* genes were cloned with 288, 330, and 275 bp of upstream sequence, respectively, to include the predicted transcriptional start sites (Seattle-King County Department of Public Health, 1984; Dugar et al., 2013; Frirdich et al., 2023). Gene expression in these strains could be driven off the predicted promoter and/or that of the chloramphenicol (Cm) resistance cassette from the pRRC plasmid (Karlyshev and Wren, 2005) upstream of the gene.

Unless otherwise indicated, *C. jejuni* strains were grown at 37°C in Mueller-Hinton (MH; Oxoid) broth or on 1.7% (w/v) agar plates supplemented with vancomycin (V; 10 μg/mL) and trimethoprim (T; 5 μg/mL), denoted MH-TV, under microaerobic/capnophilic conditions (6% O_2_, 12% CO_2_;) in a Sanyo tri-gas incubator for plates or using the Oxoid CampyGen system for broth cultures. Growth media was supplemented with Cm (C20 μg/mL) or Km (50 μg/mL), where appropriate. *Escherichia coli* strains used for plasmid construction and protein expression were grown at 37°C in Luria–Bertani (LB; Sigma) broth or 1.5% agar (w/v) agar plates unless otherwise indicated and supplemented with chloramphenicol (Cm; 20 μg/mL) or kanamycin (Km; 25 μg/mL), as necessary.

### 2.2 Pgp4, Pgp5, and Pgp6 protein expression

For expression of Pgp4, the gene encoding the recombinant Pgp4 protein was PCR amplified from *C. jejuni* 81–176 genomic DNA using primers 1,105–6 (*BspHI*) and 1,105–2 (*XhoI*) including amino acids 52–300 of the protein. For the expression of Pgp5 and Pgp6, synthetic *C. jejuni* 81–176 DNA fragments of *pgp5* and *pgp6* were codon-optimized for expression in *E. coli* (GeneWiz) and amplified from the synthetic fragments using primers optimized 1,228-a (*NdeI*) and optimized 1,228-d (*XhoI*) for *pgp5* including amino acids 20–379 of the protein, and primers optimized 0166-a (*NcoI*) and optimized 0166-b (*XhoI*) for *pgp6* including amino acids 33–457 of the protein. Primers used in this study are listed in Supplementary Table 2. The resulting PCR products were digested with restriction enzymes included in the primers and cloned into similarly digested pET28a(+) vector. *E. coli* DH5α strains were transformed with each of the recombinant plasmids and selected for on kanamycin-containing LB agar plates. The recombinant plasmids were isolated and verified by sequencing (GeneWiz).

The Pgp4, Pgp5, and Pgp6 proteins were expressed in different *E. coli* strains: C41(DE3) for Pgp4, BL21(DE3)pLysS for Pgp5, and Rosetta2(DE3)pLysS for Pgp6. The *E. coli* cells containing the respective expression plasmids were grown overnight in LB broth at 37°C. Next, 20 mL of the overnight culture was used to inoculate 1 L of 2 x YT media, which was then incubated at 37°C until the OD_600_ reached 0.8–1.0. Protein expression was induced by adding 0.25 mM IPTG, and the culture was grown at 30°C for 20 h. The cells were harvested by centrifugation at 4°C, 5,000 × g for 20 min, and the resulting pellet was either used immediately for protein purification or stored at −80°C.

### 2.3 Pgp4, Pgp5, and Pgp6 protein purification

The Pgp4 protein was purified by immobilized metal affinity chromatography on a HisPur™ cobalt resin gravity column (Thermo). The Pgp4 cell pellet was resuspended in a buffer containing 50 mM HEPES pH7.0, 300 mM NaCl, 10 mM imidazole, 1 mM phenylmethylsulfonyl fluoride (PMSF), and DNase and the cells were lysed by homogenization. The soluble fraction of the lysate was obtained by centrifugation at 15,000 rpm for 25 min and filtration through a 0.2 um filter cup and then loaded onto a HisPur™ cobalt resin gravity column previously equilibrated with equilibrate/wash buffer [50 mM HEPES pH7.0, 300 mM NaCl, 10 mM imidazole]. The column was washed with equilibration/wash buffer and the protein was then eluted with elution buffer [50 mM HEPES pH7.0, 300 mM NaCl, 150 mM imidazole]. The elution fractions containing Pgp4 were pooled, dialyzed against protein stabilization buffer containing 50 mM HEPES pH 7.0 and 300 mM NaCl, and filtered through a 0.22 um filter. The Pgp4 protein was then concentrated to 13 mg/mL and stored at −80°C.

The Pgp5 protein was purified by cation exchange chromatography with a MonoS HR 10/10 column (Cytiva) and immobilized metal affinity chromatography with a His-Trap HP column (Cytiva) using an AKTA Purifier liquid chromatography system. The Pgp5 cell pellet was resuspended in 50 mM HEPES pH 7.0, 10% glycerol, DNase, 1 mM PMSF and a protease inhibitor cocktail (Roche, cOmplete protease inhibitor cocktail EDTA-free tablet) and the cells were lysed by homogenization. The soluble fraction was obtained by centrifugation and filtration (as above). The soluble fraction of the cell lysate was loaded onto a MonoS HR 10/10 column pre-equilibrated in 50 mM HEPES pH 7.0 and 10% glycerol. The bound proteins were eluted in a 1 M NaCl gradient. To further improve the protein purity, the elution fractions containing His-tagged Pgp5 protein were loaded onto a His-Trap HP column, and washed with 50 mM HEPES pH 7.0, 500 mM NaCl, 20 mM imidazole, and 10% glycerol. The Pgp5 protein was eluted using a 400 mM imidazole gradient. The elution fractions containing the protein were then buffer exchanged via dialysis into 50 mM HEPES pH 7.0, 500 mM NaCl, and 10% glycerol, and concentrated to 4.1 mg/mL before being stored at −80°C after flash-freezing in liquid nitrogen.

The Pgp6 protein was purified by immobilized metal affinity chromatography with a His-Trap HP column (Cytiva) and size exclusion chromatography with a Superdex 75 10/300 GL column (Cytiva) using an AKTA Purifier liquid chromatography system. The Pgp6 cell pellet was resuspended in a buffer containing 50 mM HEPES pH 8.0, 300 mM NaCl, 2 mM DTT, 5% glycerol and 20 mM imidazole supplemented with DNase and a protease inhibitor cocktail (Roche, cOmplete protease inhibitor cocktail EDTA-free tablet). The cells were lysed by homogenization and the soluble fraction was obtained by centrifugation and filtration (as above). The soluble fraction was then loaded onto a His-Trap HP (Cytiva) column. Unbound proteins were washed with 20 column volumes of the binding buffer. Pgp6 was eluted with a gradient of 20–400 mM imidazole. The Pgp6 was further purified by gel filtration on a Superdex 75 10/300 GL size exclusion column using a buffer containing 50 mM HEPES pH 8.0, 300 mM NaCl, 2 mM DTT, and 5% glycerol. The fractions containing Pgp6 were pooled and the protein was then concentrated to 1 mg/mL and stored at −80°C.

### 2.4 M23 peptidase activity assay

M23 peptidase activity was assayed using PG substrates isolated from the pentapeptide-rich *E. coli* strain D456, which was isolated as previously described (Glauner, 1988). Purified recombinant Pgp4 (10 μM), Pgp5 (4 μM) or Pgp6 (4 μM) were mixed with isolated PG (~20 mg) in 20 mM HEPES/NaOH pH 7.5, 50 mM NaCl with or without EDTA (10 mM) in an assay volume of 50 μL for 4 h at 37°C. The reaction was stopped by heating at 100°C for 10 min.

The pH was adjusted to 4.8 and samples incubated overnight with 50 μg/mL cellosyl (kindly provided by Hoechst, Frankfurt, Germany). Samples were heated for 10 min at 100°C to stop the reactions, then centrifuged at 14,000 × *g* for 10 min. The supernatant was recovered and muropeptides reduced with sodium borohydride and separated by high-performance liquid chromatography as described (Glauner, 1988) using a Prontosil 120-3-C18 AQ reverse-phase column. The muropeptides were assigned using their known elution patterns and retention times (Glauner, 1988).

### 2.5 Pgp6 sequence conservation analysis

The Pgp6 homolog sequences were identified using NCBI protein blast. The input sequence CJJ81176_0166 (gene locus tag) was queried against the clustered nr database. The maximum number of hits was set to 500. The search hits were filtered based on sequence coverage to the query sequence of between 90 to 100%. The final search results identified 433 homologs as of Feb 13, 2022. A taxonomic analysis of these homologs revealed that they originated from *ε*- and *δ*-proteobacteria. Twelve distant homologs were manually selected from these taxonomic groups (Supplementary Table 3) and aligned using MUSCLE with default parameters. A phylogenetic tree was constructed using the Maximum Likelihood method with 1,000 bootstrap steps in MegaX (Kumar et al., 2018). The aligned sequences were further analyzed using Weblogo3 (Crooks et al., 2004) to plot amino acid distribution at a position in the sequence alignment.

### 2.6 Structural analysis

The structures analyzed in our study were obtained from two databases: the PDB database and the AlphaFold database. The source of each structure is described in the text. To visualize the 3D structure, we used Pymol (GLSL version 1.20) to generate cartoon graphs. Hydrogen bonds were identified based on polar contact analysis of selected residues in Pymol.

### 2.7 Microscopy and morphological analysis

For morphological analyses, *C. jejuni* strains were streaked from 16 to 18 h plate cultures and grown again on plates for 7–8 h. Bacteria were restreaked and then examined microscopically to examine morphological changes over time. Visualization under DIC microscopy was carried out as described (Frirdich et al., 2019). Quantification of the percentage of helical, coccoid and cells transitioning to the coccoid form in the DIC images was carried out by counting the number of each using Fiji image processing software. At least three separate fields of view of approximately 100–200 bacteria were counted for each strain at each time point and this was carried out for three separate cultures.

### 2.8 Phenotypic analyses: motility, biofilm formation, autoagglutination, and CFW reactivity assays

For phenotypic analyses except for autoagglutination, *C. jejuni* strains were streaked from 16–18 h plate cultures and grown again on plates for 7–8 h. Bacteria were harvested in MH-TV broth and inoculated at an OD_600_ of 0.002 into MH-TV broth and grown shaking for 18 h. Motility, biofilm formation and CFW reactivity were all carried out as described (Frirdich et al., 2012; Frirdich et al., 2014). Motility was determined from halo diameters from growth in soft agar plates (MH-TV plates containing 0.4% agar), biofilm formation assayed using crystal violet staining and CFW fluorescence by visualizing bacterial growth on agar plates containing 0.002% CFW with long wave UV. Autoagglutination assays were performed with methods adapted from Misawa and Blaser (2000). *C. jejuni* strains were streaked from 24 h plate cultures onto fresh MH-TV plates and grown overnight. Bacteria were harvested from the plates with PBS and the bacterial suspension was standardized to an OD_600_ of 1.0. 2 mL of the bacterial suspension was aliquoted to sterile glass tubes in triplicate for each time point (t = 0, 3, 6, 24 h) and incubated at room temperature. The degree of autoagglutination was quantified by measuring the OD_600_ of the top 1 mL of the bacterial suspension at each time point. The data was normalized with the OD_600_ of the bacterial suspension at t = 0 representing 100% and the OD_600_ at each time point calculated as a percentage of that at t = 0.

Mean motility and the standard error of the mean was calculated from 10 technical replicates with statistical significance determined from a one-way ANOVA with a Dunnett’s test for multiple comparisons using GraphPad Prism v.10.4.1. Data was representative of three independent experiments. The biofilm and autoagglutination assay was carried out in triplicates and is representative of three experiments. Statistical significance was calculated using a 2-way ANOVA with the Dunnett’s test for multiple comparisons using GraphPad Prism v.10.4.1.

### 2.9 Hydrophobicity

Two methods of determining cell surface hydrophobicity were used: the salt aggregation test (SAT) and the bacterial adhesion to hydrocarbons (BATCH). SAT was carried out as described by Misawa and Blaser (2000) with plate grown bacteria as for the autoagglutination test. The well with bacteria with 2 mM sodium phosphate only served as the negative control and that with an initial concentration of 4 M ammonium sulfate in 2 mM sodium phosphate as the positive control. BATCH was carried out as described by Ha et al. (2016) with overnight cultures prepared as above.

### 2.10 pH sensitivity testing

The pH sensitivity was assessed on agar plates. The plates were prepared by separately autoclaving the agar and broth with the pH of the MH broth being adjusted with concentrated HCl before autoclaving. After autoclaving, the agar and broth were combined and the TV was added. Overnight cultures were prepared as above except they were inoculated at an OD_600_ of 0.002 for the wild type, OD_600_ of 0.001 for the mutant and OD_600_ of 0.008 for the complemented and overexpression strains to adjust for differences in growth rate. The overnight cultures were standardized to an OD_600_ of 0.50 OD/mL and serially diluted 10-fold in MH-TV broth in a microtiter plate. 5 μL of each dilution was spot plated on MH-HCl-TV plates at pH 4.5, 5.0, 5.5, 6.0, 6.5, 7.0, and MH-TV unadjusted for pH (approximately pH 7.1). The MH-TV plates were incubated for 2 days at 37°C under microaerophilic conditions to assess growth at each dilution. The experiment was carried out with biological triplicates.

### 2.11 Antimicrobial sensitivity

The susceptibility of *C. jejuni* strains to different compounds was determined by a standard microtiter broth dilution method as previously described (Sahm and Washington II, 1991). Briefly, overnight cultures prepared as above were standardized to an OD_600_ of 0.0002 OD/mL (10^6^ cfu/mL) in MH media (approximately pH 7.1) or MH media adjusted to pH 5.0 with HCl. 100 μL of the bacterial suspension was added to each well of serial doubling dilutions of 11 μL of concentrated test compound at 10x the required concentration. For cationic antimicrobial peptides (polymyxin B and protamine), adjustments were made as described.^1^ Microtiter plates were incubated at 37°C without shaking for 2 days under microaerophilic conditions. Then 5 μL of each well was spot plated onto MH plates and grown for an additional 2 days at 37°C under microaerophilic conditions to assess growth. The MIC_50_ was defined as the lowest concentration of compound that reduced growth by 50%. The experiment was carried out with biological triplicates.

### 2.12 *In vitro* adherence, invasion and intracellular survival in epithelial cells

A gentamicin protection assay was used to assess *C. jejuni in vitro* adherence, invasion and intracellular survival in the human epithelial INT407 cell line as described (Frirdich et al., 2012; Frirdich et al., 2014). The INT407 cell line was obtained from the American Type Tissue Culture Collection (ATCC; ATCC CCL-6). Briefly, INT407 cells were seeded into 24-well tissue culture plates at semiconfluence at approximately 1 × 10^5^ cells/ml and allowed to grow for 20–24 h prior to infection. Infections were initiated with approximately 1 × 10^7^ CFU/mL of *C. jejuni* from an 18 h shaking broth culture (t = 0 representing the inoculum). Infections were carried out for 3 h (t = 3 adhered and invaded time point). Gentamicin (250 μg/mL) was added 3 h post infection and incubated for 2 h (t = 5 invasion time point). After 2 h, the gentamicin was washed off, and the cells were incubated with fresh MEM containing 3% FBS and a low dose of gentamicin (10 ug/mL) for an additional 2 h (t = 7 intracellular survival time point). Cfu/mL were determined for each well by lysing the cells with water and plating the dilutions onto MH-TV plates. Standard errors of the mean were calculated from triplicate readings and are representative of three independent experiments. The data was normalized with the cfu of the inoculum (t = 0) representing 100% and the cfu at each time point calculated as a percentage of the inoculum. Statistical significance was calculated using a 2-way ANOVA with the Dunnett’s test for multiple comparisons using GraphPad Prism v.10.4.1.

## 3 Results

### 3.1 Functional analysis of Pgp4, Pgp5, and Pgp6

#### 3.1.1 Substrate specificity of Pgp4, Pgp5, and Pgp6

The biochemical activity of Pgp4, Pgp5 and Pgp6 was determined in a similar manner to the method used for Pgp1 and Pgp2. Each protein was expressed, purified and incubated with purified PG. The resultant muropeptide profile was examined by HPLC and compared to that of the PG without added enzyme to determine the activity of the enzyme.

**Figure 1:**
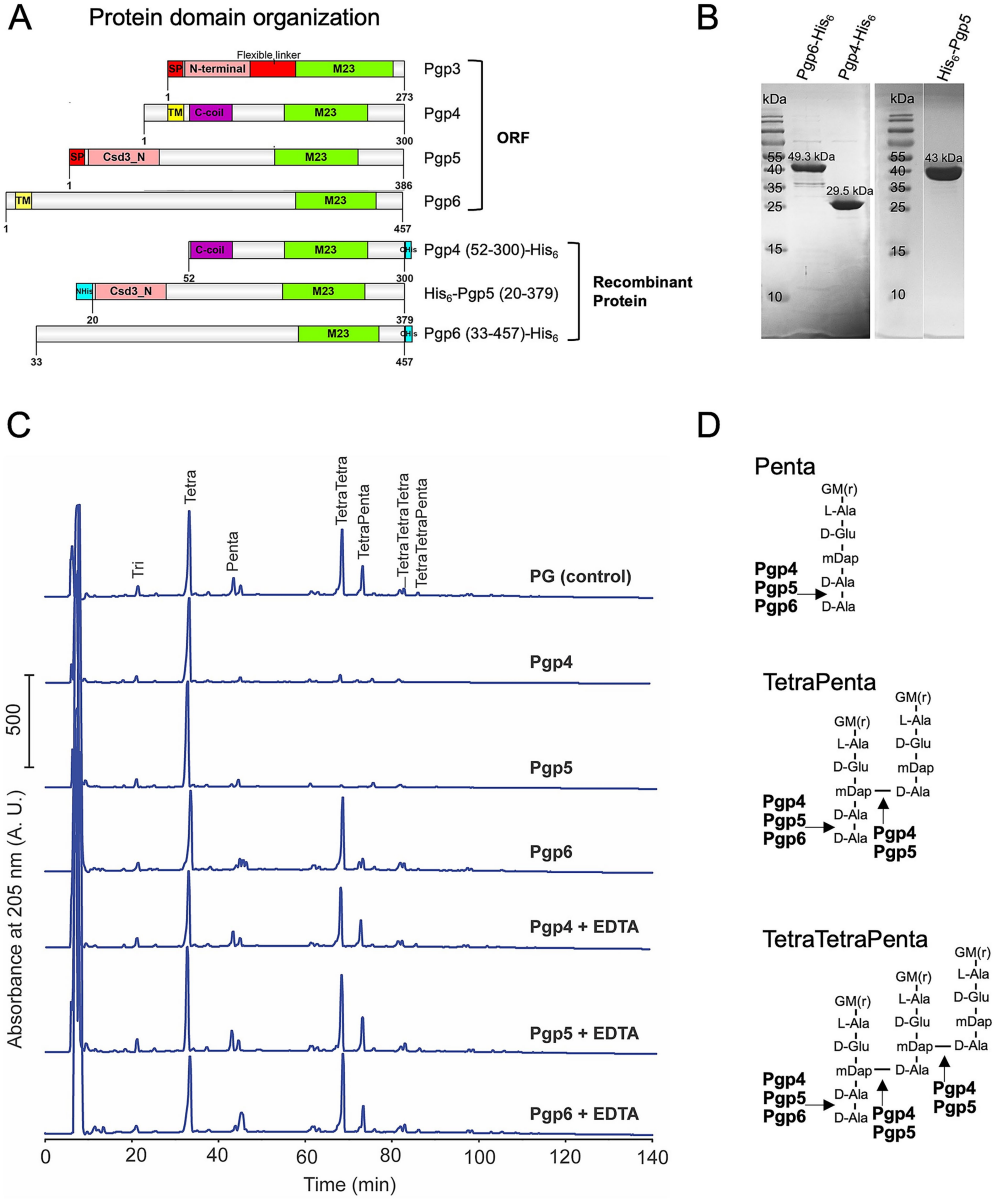
*In vitro* DD-EPase and DD-CPase activity of Pgp4 and Pgp5, and DD-CPase activity of Pgp6. **(A)** protein domain organization of *C. jejuni* M23 peptidases Pgp3, Pgp4, Pgp5 and Pgp6 and the recombinant proteins used for protein expression and purification. The Pgp4, Pgp5, and Pgp6 domains were predicated by jackhammr (Frirdich et al., 2023) and Pgp3 domains were assigned based on the available crystal structure (Min et al., 2020). **(B)** Purified recombinant Pgp4, Pgp5 and Pgp6 proteins separated on 12% SDS-PAGE and stained with Coomassie Brilliant Blue. The predicted molecular weight of each protein is indicated above the band. **(C)** HPLC chromatograms of *E. coli* D456 PG incubated with purified Pgp4, Pgp5 and Pgp6 with and without EDTA. *E. coli* D456 PG alone was used as a control. **(D)** Schematic diagram of the muropeptide substrates and the Pgp4, Pgp5 and Pgp6 cleavage sites indicated with arrows. G, N-acetylglucosamine; M(r), reduced N-acetylmuramic acid; L-Ala, L-alanine; D-Glu, D-glutamic acid; mDAP, meso-diaminopimelic acid; D-Ala, D-alanine.

Bioinformatic analyses predicted that the three *C. jejuni* M23 enzymes contain a C-terminal M23 peptidase domain but display various protein domain organizations in the N-terminal region (Figure 1A): Pgp4 has a transmembrane (TM) domain and a coiled-coil region, Pgp5 contains a predicted signal peptide and a Csd3_N domain, and Pgp6 has a TM domain followed by an unknown region. The TM domain and signal peptide play a role in cellular localization in *C. jejuni*. Protein purification constructs were designed such that the TM domains (Pgp4 and Pgp6) and signal peptide (Pgp5) were not included to ensure cytoplasmic *E. coli* expression (Figure 1A). The disordered region was removed from the C-terminus of Pgp5 (amino acids 380–386) to increase protein expression levels (Figure 1A). A His_6_-tag for protein purification was added to both the N- and C-terminus, with the construct resulting in the highest protein expression levels being selected (C-terminus for Pgp4 and Pgp6 and N-terminus for Pgp5; Figure 1A). The recombinant proteins were expressed in *E. coli* and purified to >90% purity as determined by SDS-PAGE (Figure 1B). Since these M23 peptidases were predicted to have DD-CPase and/or DD-EPase activity, pentapeptide enriched PG isolated from *E. coli* strain D456, a triple DD-CPase deletion mutant was used as the substrate (Edwards and Donachie, 1993; Potluri et al., 2010). Purified Pgp4, Pgp5 or Pgp6 was incubated with *E. coli* D456 PG and the resulting muropeptide profile was examined by HPLC and compared to a no enzyme control (Figure 1C). The addition of Pgp4, Pgp5, and Pgp6 resulted in the cleavage of the terminal D-Ala residue of pentapeptide containing muropeptides (Penta, TetraPenta, and TetraTetraPenta species) demonstrating DD-CPase activity. In addition, no cross-linked muropeptides were detected in the PG of samples to which Pgp4 and Pgp5 were added, indicating that Pgp4 and Pgp5 also had DD-EPase activity cleaving 4–3 cross-links. While formally possible, it is highly unlikely that contaminating *E. coli* DD-EPases MepA and MepM contributed to the observed activities as we did not detect significant DD-EPase activity with Pgp6 and detected DD-CPase activity for Pgp4 and Pgp6, which cannot be provided by possible contaminating *E. coli* DD-EPases.

The majority of the M23 peptidase domain proteins contain a characteristic zinc-binding motif H(x)_n_D, HxH and are zinc-dependent metallopeptidases (Razew et al., 2022). To examine whether the metal is required for Pgp4, Pgp5, and Pgp6 catalysis, we examined the enzyme activity without metal by adding the metal chelator EDTA. All three enzymes lost activity in the presence of EDTA with the muropeptide peptide profiles being identical to that of the PG only control (Figure 1C). In summary, Pgp4, Pgp5, and Pgp6 are metallopeptidases with Pgp4 and Pgp5 having DD-CPase and DD-EPase activity similar to that of the other known *C. jejuni* M23 enzyme Pgp3 (Min et al., 2020), while Pgp6 only has DD-CPase activity (Figure 1D).

#### 3.1.2 The M23 DD-CPase Pgp6 has four insertion sequences (SEQ1-4) not present in the M23 domain of Pgp3

M23 peptidase domains are zinc metallopeptidases and are common PG hydrolases with various substrate specificities (Razew et al., 2022). For instance, *S. aureus* LytM is a DD-EPase that cleaves the Gly-Gly bond of pentaglycine cross-bridges in the Gram-positive cell wall (Firczuk et al., 2005); *H. pylori* Csd1 (An et al., 2016) (note Csd1 functional activity was determined from the muropeptide profile of the mutant and not by biochemical activity assay) and *V. cholerae* ShyA (Shin et al., 2020) are DD-EPases that cleave between D-Ala and mDap of 4–3 cross-links in Gram-negative PG; and some enzymes such as *H. pylori* Csd3 (An et al., 2015), *C. jejuni* Pgp3 (Min et al., 2020), and, as shown in this study, Pgp4 and Pgp5 have both DD-EPase and DD-CPase activity and cleave the D-Ala and mDap bond of 4–3 cross-links and the terminal D-Ala from the pentapeptide. M23 peptidase domain proteins with only DD-CPase activity have not been identified as of yet (see Razew et al. (2022) for a list of enzyme activities of characterized M23 peptidases). We hypothesized that Pgp6 may have a functional motif in its M23 fold to restrict it to monomeric pentapeptides.

**Figure 2:**
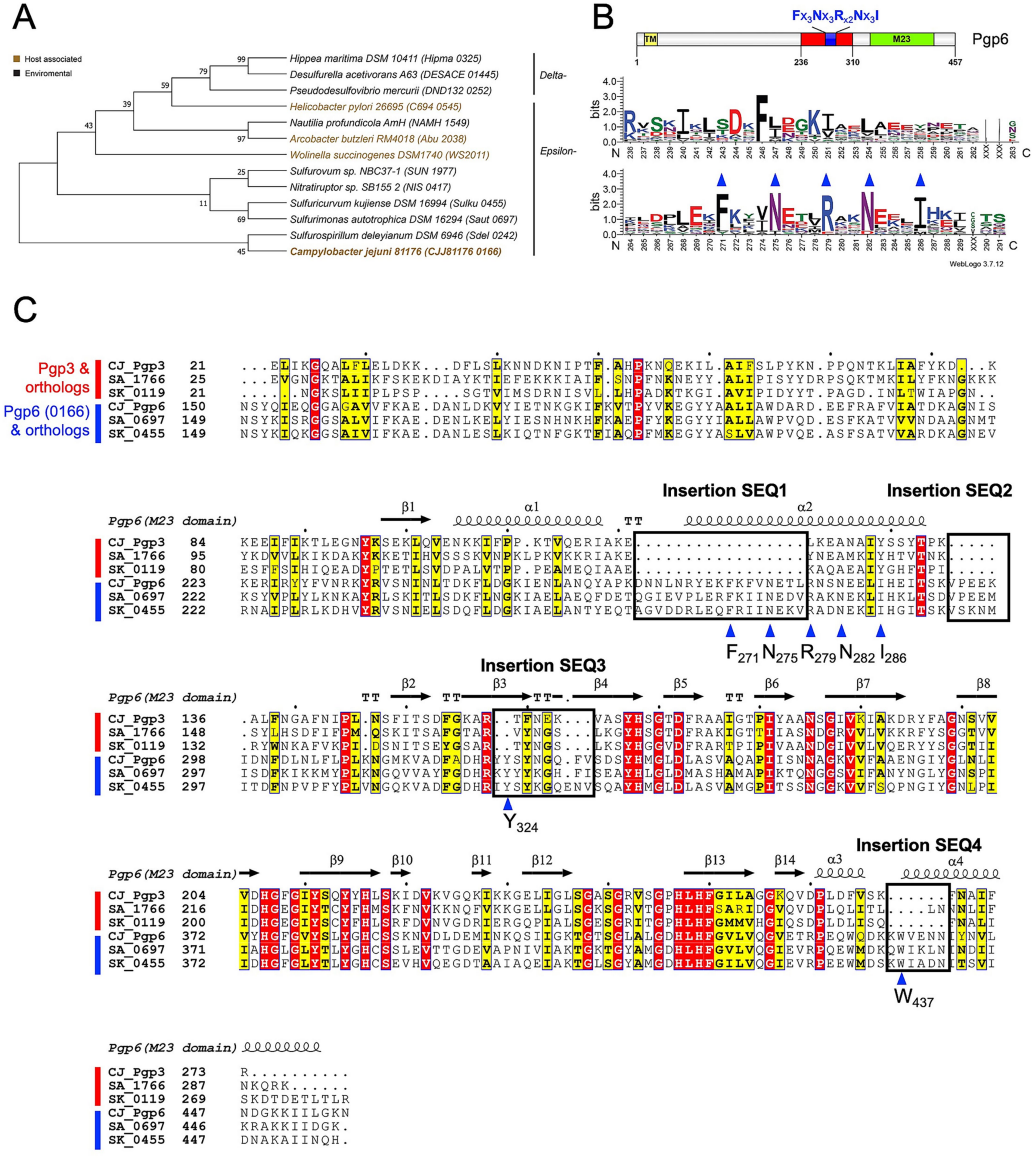
Pgp6 sequence analysis. **(A)** Phylogenetic tree representing Pgp6/0166 and its orthologs used for conservation analysis. Sequences are designated by the species name and the gene locus tag in brackets. The tree was constructed from a multiple sequence alignment of Pgp6 and its orthologs using the maximum likelihood method in MegaX. **(B)** Sequence logo constructed using Weblogo3 (Crooks et al., 2004) representing the amino acid distribution at a position created from the alignment used in **(A)**. The numbering on the x-axis corresponds to the residue number in Pgp6. The graph indicates a consensus sequence Fx_3_Nx_3_Rx_2_Nx_3_I before the start of the M23 peptidase domain. **(C)** Multiple sequence alignment of Pgp3, Pgp6, and their orthologs (shown by gene locus tag) from *Sulfurimonas autotrophica DSM 16294* (SA) and *Sulfuricurvum kujiense DSM 16994* (SK) plotted using ENDscript 3.0 (Robert and Gouet, 2014). Four insertion sequences (SEQ1-4), boxed and labeled, are unique to Pgp6. The blue triangle indicates a conserved residue within or close to the insertion sequences.

Amino acid positions making up sites of structural or functional importance in a protein are evolutionarily conserved and evolve more slowly than variable positions (Ashkenazy et al., 2010). Therefore, we searched for a highly conserved region in Pgp6 that may indicate a functional site selecting for the monomeric pentapeptide substrate and not the crosslinked substrate by performing a sequence alignment with Pgp6 and its orthologs. Using NCBI blastp,^2^ we found Pgp6 orthologs distributed in the *ε*- and *δ*-proteobacteria in the clustered nr database. We manually selected 12 orthologs (query coverage between 92 to 99%; sequence identity between 27 to 46%; and E-value between 5e-151 to 1e-43) (Figure 2A) and used MUSCLE (Edgar, 2004) to align them. The amino acid distribution at each position of Pgp6 orthologs was plotted using WebLogo3 (Crooks et al., 2004). A consensus sequence (Fx_3_Nx_3_Rx_2_Nx_3_I) was identified in Pgp6 before the start of the M23 peptidase domain (Figure 2B).

We searched for the consensus sequence (Fx_3_Nx_3_Rx_2_Nx_3_I) in Pgp3, Pgp4, and Pgp5, but it was found to be unique to Pgp6. Pgp6 shares higher sequence similarity to Pgp3 (37.40% identity/ 2e-21 E-value) than to Pgp4 (23.6% identity/4e-14 E-value) and Pgp5 (28.2% identity/2e-14 E-value). Therefore, a sequence alignment of Pgp3, Pgp6, and their orthologs from *Sulfurimonas autotrophica DSM 16294* and *Sulfuricurvum kujiense DSM 16994* was performed (Figure 2C). This suggested that Pgp6 aligns well with Pgp3 except for four insertion sequences: SEQ1(D262-L278), SEQ2 (V293-I298), SEQ3 (Y323-V331), and SEQ4 (K436-N441) (Figure 2C). SEQ1 encompasses part of the consensus sequence (Fx_3_Nx_3_Rx_2_Nx_3_I). SEQ3 and SEQ4 contain conserved residues Y324 and W437. The amino acid conservation and its unique occurrence in Pgp6 suggest that these insertion sequences may play a role in limiting the activity of Pgp6 to monomeric substrates, as opposed to Pgp3 that can act on monomeric and dimeric substrates.

#### 3.1.3 The Pgp6 AlphaFold model suggests that the insertion sequences SEQ1-4 restrict the active site groove to smaller substrates

**Figure 3:**
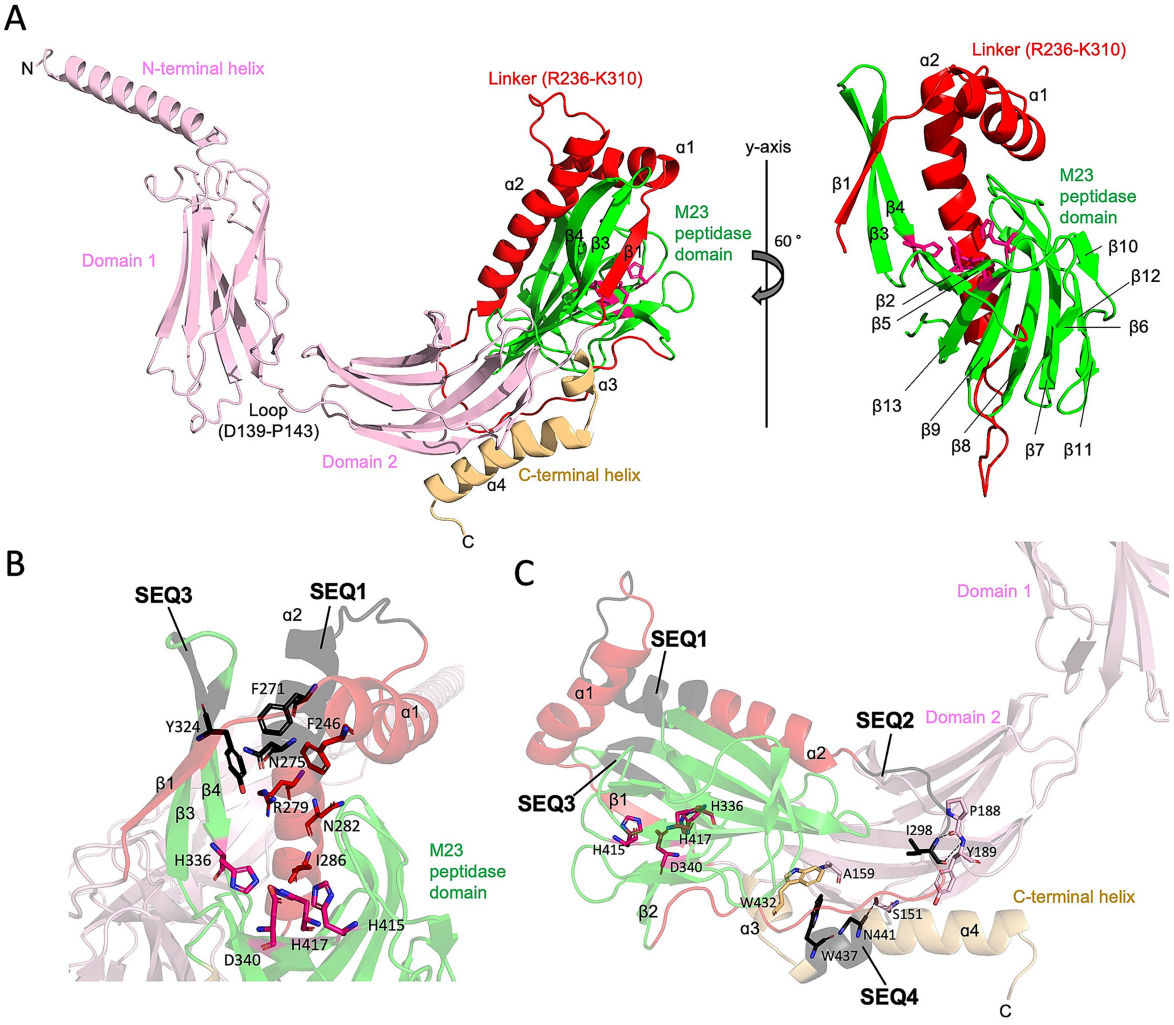
The AlphaFold model of Pgp6 and insertion sequences (SEQ1-SEQ4). **(A)** Ribbon diagram of the Pgp6 AlphaFold model. The N-terminal helix, domain 1, and domain 2 are shown in pink, the linker between domain 2 and M23 peptidase domain in red, the M23 peptidase domain in green, and the C-terminal helix in light orange. Residues of the Zn binding motif (H336, D340, H415, and His417) are shown in stick format. The secondary structure elements of the linker and M23 peptidase domain are labeled. **(B)** Zoomed in view of insertion sequences SEQ1 and SEQ3. The insertion sequences are shown in black. Side chains of residues in the consensus sequence Fx_3_Nx_3_Rx_2_Nx_3_I (F271, N275, R279, N282, and I286) are facing toward the catalytic Zn binding motif. Aromatic residues F271 in SEQ1 and Y324 in SEQ3 cluster with a conserved residue F246. **(C)** Diagram of the partial Pgp6 model showing that SEQ2 and SEQ4 form contacts that anchor the M23 peptidase domain to domain 2. The I298 residue in SEQ2 is hydrogen bonded to the main chain of P188 and Y189; N441 in SEQ4 is hydrogen bonded to the hydroxyl group of S151; and W437 in SEQ4 forms a hydrophobic core with residues W432 and A159. Hydrogen bonds are indicated as dash lines.

To gain more insight into the influence of the insertion sequences on Pgp6 function, we examined the position of the insertion sequences in the Pgp6 protein structure. As there is no available experimental structure for Pgp6, we retrieved a computational model from the Alpha Fold database (Jumper et al., 2021; Varadi et al., 2022). The Pgp6 model (AlphaFold ID A0A0H3PIR6) (Figure 3A, left) had an N-terminal helix (M1-R27), followed by two immunoglobulin-like *β*-sandwich folds: domain 1 (L28-V138) and domain 2 (K144-Y235), and an M23 peptidase domain (N311-P429). Between domain 1 and domain 2 is a short loop (D139-P143). Domain 2 is connected to the M23 peptidase domain by a linker region (R236-K310) consisting of strand β1 and helices ɑ1ɑ2 (See protein topology of Pgp6 M23 peptidase domain in Supplementary Figure 2). The C-terminal helix (E430-N457) folds back on domain 2. The model consisting of domain 2 to the C-terminal helix (N140 to N457) is predicted to be of high confidence by AlphaFold (Supplementary Figure 3). The per-residue confidence score (pLDDT) is above 96 (this score is between 0 and 100, with 100 meaning the highest confidence) (Jumper et al., 2021). Predicated aligned error (PAE), which represents the distance error (Å) of 2 residues, is low (between 0 and 15 Å), suggesting the relative position and orientation between domains are well defined (Varadi et al., 2022).

**Figure 4:**
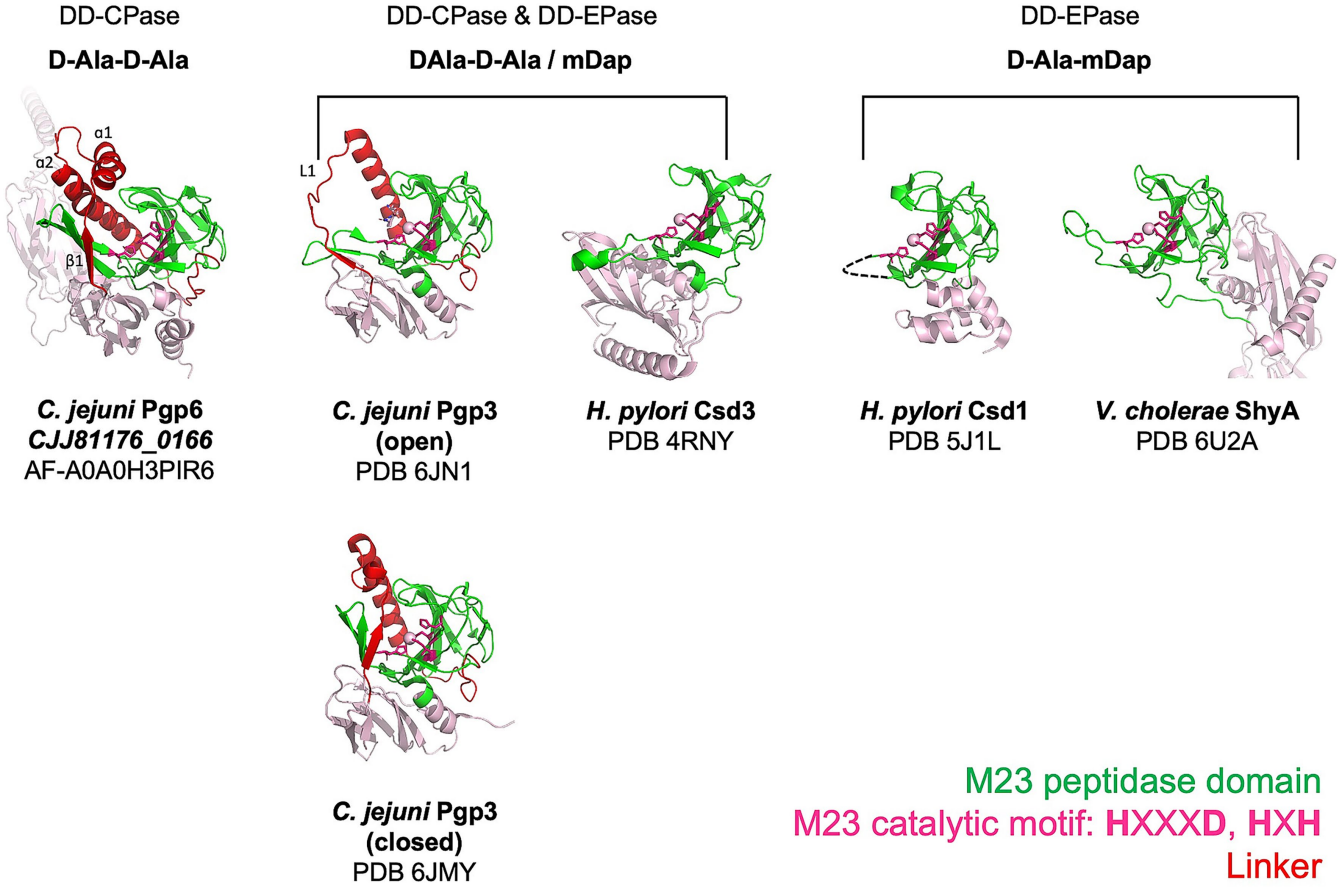
Comparison of M23 peptidase domain proteins with different PG substrates. Ribbon diagrams showing the architecture of M23 peptidase domains from enzymes that cleave the terminal D-Ala of pentapeptide side chains and/or the 4-3 cross-links. Pgp6 is a predicted structure retrieved from the AlphaFold database. *C. jejuni* Pgp3 (Min et al., 2020), *H. pylori* Csd3 (An et al., 2015), *H. pylori* Csd1 (An et al., 2016), and *V. cholerae* ShyA (Shin et al., 2020) are experimental structures obtained by X-ray crystallography. The M23 peptidase domain is shown in green, the linker that connects the N-terminal region and the M23 peptidase domain in red, and the remainder of the model in pink. The zinc coordinating residues are shown as stick models, and the zinc metal ion, if available, is drawn as a sphere. The functional activity and the identity of the amino acids of the peptide substrate of each enzyme are written above the enzyme models. Note the functional activity of Csd1 was determined from the muropeptide profile of the mutant and not by biochemical activity assay.

The Pgp6 model shows that helices ɑ1 and ɑ2 form a helix–loop–helix structure that is situated between the core β-sheet, which consists of seven strands (β2, β5, β13, β9, β8, β7, and β11) and another β-sheet of strands made up of β1, β3 and β4 (Figure 3A, right). The insertion sequences SEQ1 and SEQ3 extend the length of helix ɑ2 as well as strands β3 and β4, respectively. This results in an enclosed surface that wraps the top and one side of the concave surface in the M23 peptidase domain, creating a single-entry point for the substrate (Figure 3B). The β-ɑ-β sandwich is stabilized by numerous hydrophobic contacts, including a hydrophobic cluster formed by residue F271 in SEQ1, Y324 in SEQ3, and a conserved residue F246 in ɑ2 (Figure 3B). In contrast, the equivalent position of ɑ1ɑ2 helix pair in the Pgp3 enzyme with DD-EPase and DD-CPase activity is a flexible linker. Loop L1 of Pgp3 can adopt a huge conformational change to switch from a latent conformation (named as closed form) to a substrate binding conformation (named as open form) (Figure 4) (Min et al., 2020). A comparison of the active site groove between Pgp6 and the open conformation of Pgp3 suggests that Pgp6 has a small substrate entry access, which may limit the conformation and types of substrates. The side chains of Fx_3_Nx_3_Rx_2_Nx_3_I in SEQ1 of Pgp6 face toward the zinc binding ligands (Figure 3B). The R279 residue is only 7.5 Å from the zinc binding ligands, suggesting that the positively charged side chain is in a good position to orient the negatively charged PG substrate (e.g., carboxylate group of the terminal D-Ala of the pentapeptide).

The insertion sequences SEQ2 and SEQ4 are remote from the zinc binding center (Figure 3C). SEQ2 belongs to the ɑ2-β2 loop adjacent to a loop (F184-Y194) in domain2. The amide nitrogen and main chain oxygen of residue I298 in SEQ2 are hydrogen-bonded to the main chain of residues P188 and Y189 in domain 2 (Figure 3C). SEQ4 is located at the C-terminal helix, and it participates in the contacts between the M23 peptidase domain and domain 2. The main chain oxygen of residue N441 in SEQ4 makes a hydrogen bond to the side chain hydroxyl group of Ser151 in domain 2; residue W437 has a hydrophobic interaction with the side chain methyl group of Ala159 in domain 2 and a conserved residue W432 (Figure 3C). In summary, from the AlphaFold model of Pgp6, the insertion sequences SEQ1-4 contribute additional contacts within the M23 peptidase domain and between domains with domain 2. We propose that Pgp6 only has DD-CPase activity because of the small active site groove created by the insertion sequences SEQ1 and SEQ3 that selects for the monomeric pentapeptide. The contacts made by SEQ2 and SEQ4 appear to help generate the interdomain orientation of domain 2 and the core of the M23 peptidase domain.

### 3.2 The role of Pgp4, Pgp5, and Pgp6 in *C. jejuni* biology and pathogenesis

#### 3.2.1 The effects of *pgp4, pgp5,* and *pgp6* on *C. jejuni* biological properties

**Figure 5:**
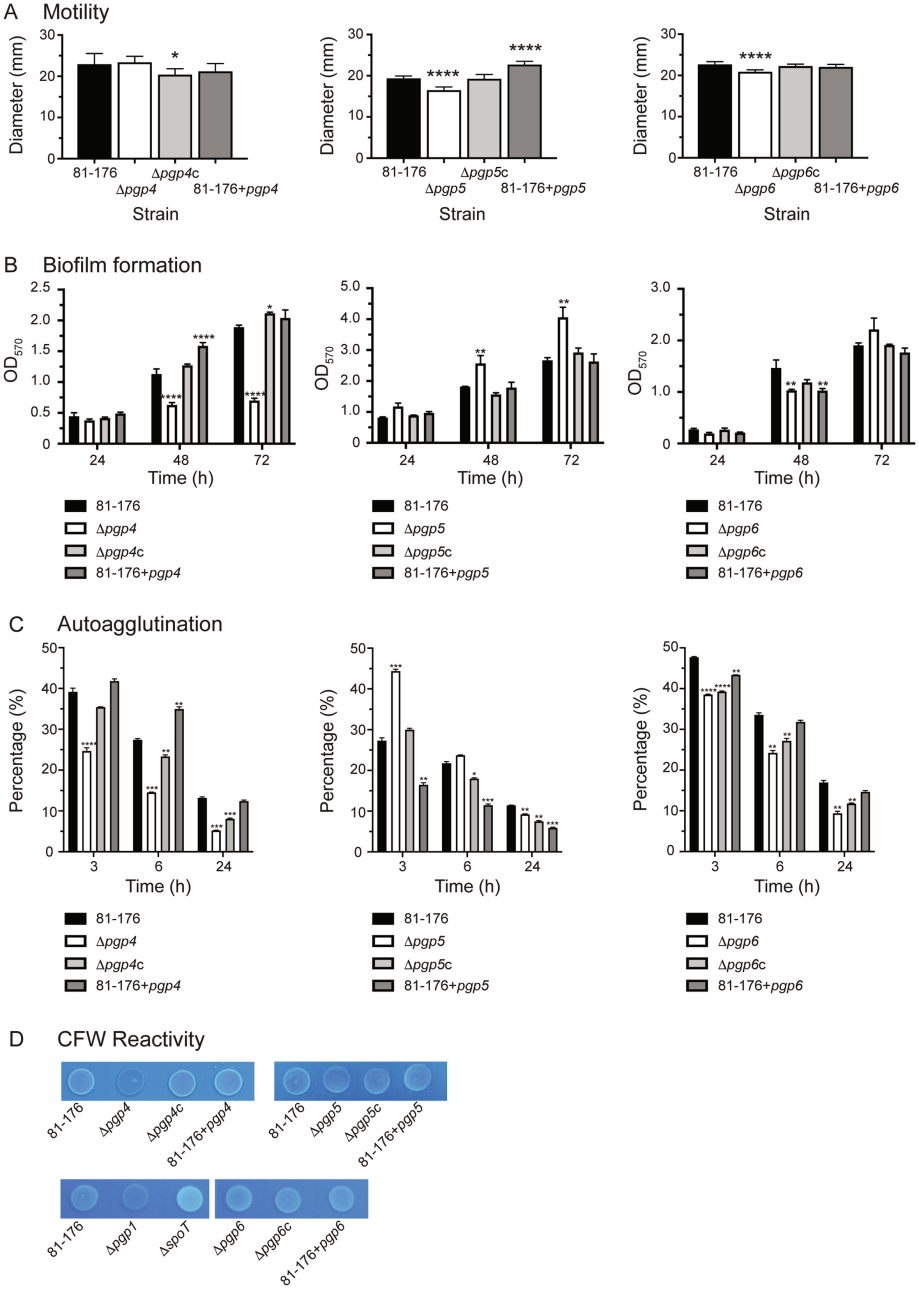
The effect of *pgp4, pgp5* and *pgp6* on motility, biofilm formation, autoagglutination and CFW reactivity. The phenotypic properties of the *pgp4*, *pgp5* and *pgp6* mutant, complemented (∆*pgp4c*, ∆*pgp5c*, and ∆*pgp6c*) and overexpression (81–176 + *pgp4*, 81–176 + *pgp5* and 81–176 + *pgp6*) strains were assessed. **(A)** Motility assayed by measuring halo diameters in soft agar plates. S.E. (error bars) was calculated from 10 technical replicates. Statistical significance was determined using a one-way ANOVA with a Dunnett’s test for multiple comparisons. Data was representative of three independent experiments **(B)** biofilm formation assessed by crystal violet staining of standing cultures in borosilicate tubes and quantification of dissolved crystal violet at 570 nm. S.E. values were calculated from triplicate cultures and are representative of three independent experiments. Statistical significance was determined using a two-way ANOVA with a Dunnett’s test for multiple comparisons. **(C)** Autoagglutination was measured at 0, 3, 6, and 24 h in PBS at 25°C. The data was normalized with the OD_600_ at t = 0 representing 100% and the OD600 at each timepoint calculated as a percentage of that at t = 0. A decrease in percentage represents an increase in autoagglutination. S. E. values were calculated from triplicate cultures and are representative of three independent experiments. Statistical significance was determined using a two-way ANOVA with a Dunnett’s test for multiple comparisons. **(D)** The fluorescence relative to wild type after 48 h of growth on plates containing 0.002% CFW. The controls included ∆*spoT* and ∆*pgp1* representing a hyperfluorescent and hypofluorescent strain, respectively. Note that in the bottom row, all strains are compared to the wild type shown in the first panel of that row as both panels are cropped from the same image. The asterisk (*) indicates a statistically significant difference in comparison to wild type, with *, **, ***, or *** indicating *p* < 0.05, *p* < 0.01, *p* < 0.001 and *p* < 0.0001, respectively.

Motility was assayed by measuring halo diameter in soft agar plates (Figure 5A). No flagellar structural defects were observed by electron microscopy (Frirdich et al., 2023). The motility of ∆*pgp4* was similar to wild type. However, the complemented strain (*∆pgp4c*) showed a statistically significant but slight decrease in motility of 11.1% in comparison to wild type and the overexpression strain (81–176 + *pgp4*) a decrease of 7.6% that was not statistically significant. The ∆*pgp5* strain displayed a motility defect which was similar to that reported previously (85.1% of wild type motility) (Stahl et al., 2016). The complement restored motility to wild type levels, while the *pgp5* overexpression strain showed an increase of motility of 17.2% in comparison to wild type. The motility of the ∆*pgp6* strain was slightly decreased by 7.9% in comparison to wild type. Complementation restored motility to wild type levels with the *pgp6* overexpression strain showing a very slight increase in motility of 2.9% in comparison to wild type.

Biofilm formation was assessed by crystal violet assays and measured over 3 days (Figure 5B). Biofilm levels of ∆*pgp4* were similar to wild type at day 1 and then 1.8-, and 2.7-fold lower at days 2 and 3, respectively. Biofilm production was restored in the complemented strain (∆*pgp4c*) and slightly above wild type by day 3. The *pgp4* overexpression strain produced higher levels of biofilm than wild type at day 2 but not day 3. Biofilm levels of ∆*pgp5* were 1.5-fold higher than wild type at days 2 and 3. Wild type biofilm levels were seen in the complemented and *pgp5* overexpression strain. Biofilm levels of ∆*pgp6* were 1.4-fold lower than wild type at day 2 and then 1.2-fold higher at day 3 (although this increase was not significant). The complement showed wild type biofilm levels with the *pgp6* overexpression displaying 1.4-fold lower than wild type levels at day 2 but not day 3.

*C. jejuni* autoagglutination is a measure of virulence. The autoagglutination kinetics were determined by monitoring the levels of autoagglutination at 3, 6, and 24 h in PBS at room temperature (Figure 5C). The ∆*pgp4* and ∆*pgp6* mutants showed higher levels of autoagglutination than wild type at 3, 6 and 24 h. This was not complemented to wild type levels in ∆*pgp4c* and ∆*pgp6c* (except for ∆*pgp4c* at 3 h). The *pgp4* overexpression strain displayed decreased autoagglutination at 6, while the *pgp6* overexpression strain displayed slightly increased autoagglutination at 3 h. The ∆*pgp5* mutant autoagglutination was less than wild type at 3 h but was slightly increased in comparison to wild type by 24 h. The ∆*pgp5* complemented strain showed increased autoagglutination in comparison to wild type at 6 h and 24 h with the levels being higher than the ∆*pgp5* mutant at 24 h. The *pgp5* overexpression strain had an even more pronounced increase in autoagglutination than ∆*pgp5*c at 3 h, 6 h and 24 h.

No difference in hydrophobicity was detected between the strains by either the SAT or BATCH method (data not shown) of examining hydrophobicity. Using the SAT method, all strains precipitated/aggregated in an initial ammonium sulfate concentration of 0.0313 M.

CFW fluorescence was determined by visualizing bacterial growth on agar plates containing CFW under long wave UV (Figure 5D). After 48 h of growth, the ∆*pgp1* strain is hypofluorescent and the ∆*spoT* strain hyperfluorescent in comparison to wild type and are used as controls. The ∆*pgp4* strain was hypo-fluorescent, while the complement and *pgp4* overexpression strain were similar to wild type. Despite displaying an increase in biofilm formation, ∆*pgp5* showed wild type CFW fluorescence, as did ∆*pgp5*c and the *pgp5* overexpression strain. The ∆*pgp6*, ∆*pgp6*c and *pgp6* overexpression strains all showed wild type fluorescence.

**Figure 6:**
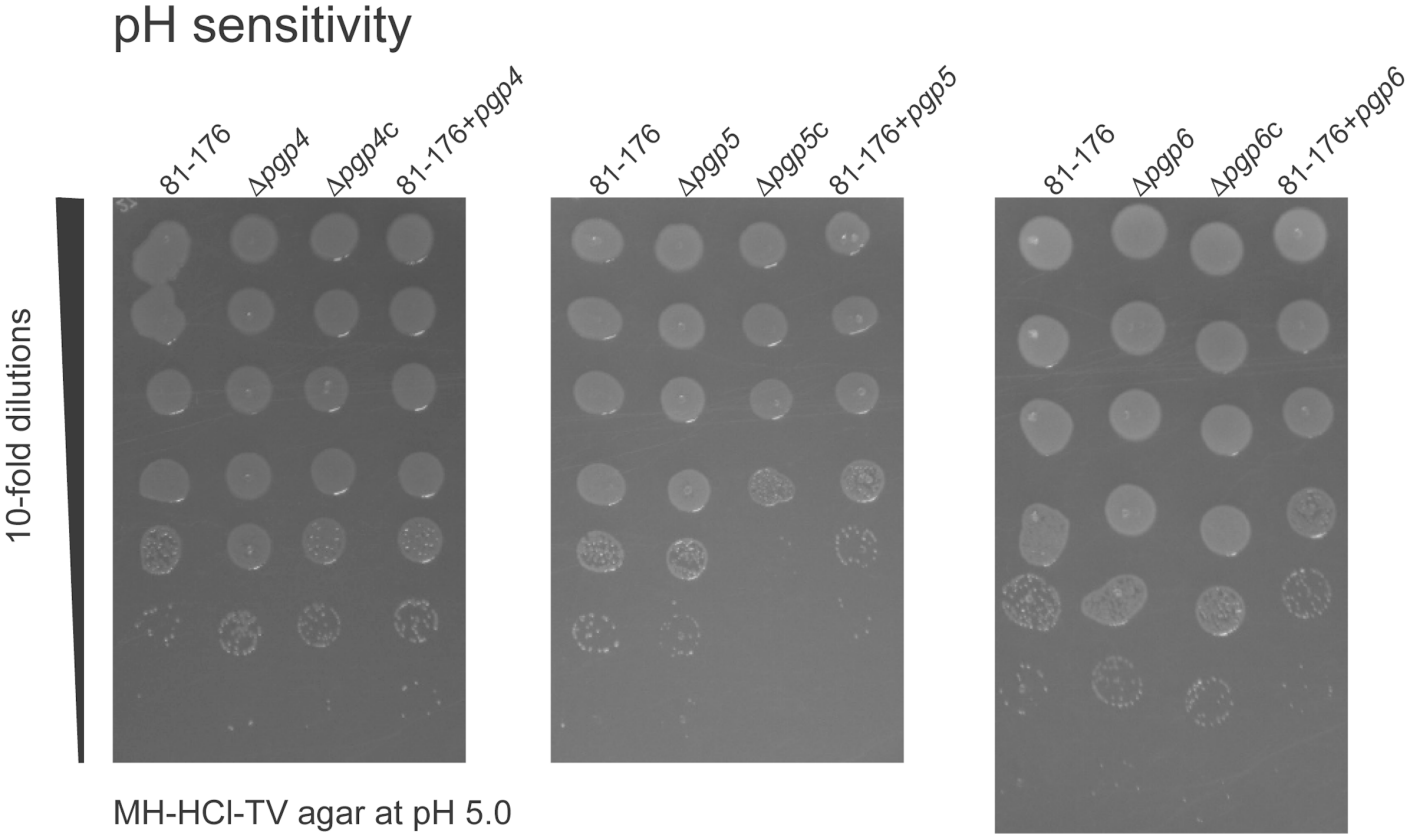
The effect of *pgp4, pgp5* and *pgp6* on acid survival. The ability of the *pgp4*, *pgp5* and *pgp6* mutant, complemented (∆*pgp4c*, ∆*pgp5c*, and ∆*pgp6c*) and overexpression (81–176 + *pgp4*, 81–176 + *pgp5* and 81–176 + *pgp6*) strains to grow on MH-HCl-TV agar at pH 4.5, 5.0, 5.5, 6.0, 6.5, 7.0 and MH-TV unadjusted for pH was assessed. Only growth on pH 5.0 showed differences in comparison to wild type and is shown here. Overnight cultures were standardized to an OD_600_ of 0.50 OD/mL and serially diluted 10-fold in MH-TV broth in a microtiter plate. 5 μL of each dilution was spot plated with the most concentrated at the top of the plate. The MH-HCl-TV plates were incubated for 2 days at 37°C under microaerophilic conditions to assess growth at each dilution. The data presented is representative of three independent experiments.

Some PG hydrolases are preferentially used by the cell under certain growth conditions such as acidic pH (Egan et al., 2020). Therefore, we wanted to determine whether one of Pgp4, Pgp5 or Pgp6 was more important for growth at low pH by assessing acid survival. The ability of the *pgp4*, *pgp5* and *pgp6* mutant, complemented and overexpression strains to survive on MH-TV agar plates at pH 4.5, 5.0, 5.5, 6.0, 6.5 and 7.0 was compared to that of wild type. At pH 4.5, there was very little to no growth. A difference in survival of the strains was only seen at pH 5.0 so only the results from that pH are shown (Figure 6). At pH 5.0, the survival of ∆*pgp5c*, 81–176 + *pgp5*, and 81–176 + *pgp6* strains was reduced at least 10-fold in comparison to wild type. pH survival experiments carried out in broth produced inconsistent results and were not included.

In order to assess the integrity of the outer membrane of ∆*pgp4*, ∆*pgp5*, ∆*pgp6*, the minimum inhibitory concentration that reduced growth by 50% of the mutants to detergents, antimicrobial compounds, chelating agents and salts was determined and compared to that of wild type. There were no large differences in sensitivity of the mutants in comparison to wild type (Supplementary Table 4). Interestingly, the MIC to MES (2-(N-morpholino) ethanesulfonic acid) decreased at pH 5.0 in comparison to pH 7.0. MES is used as a buffering agent with a buffering capacity of 5.5–7.0 due to its good solubility in water, high stability and minimal interference with biological compounds. Our studies examining pH survival in MH media made up with 50 mM and 100 mM MES as a buffering agent indicated that MES may be having an effect on *C. jejuni* survival which was confirmed by determining the *C. jejuni* MIC to MES. Because of the decrease in MIC for MES at pH 5.0, the MIC of all compounds tested was determined at pH 5.0 (Supplementary Table 4). Interestingly, the MIC at pH 5.0 in comparison to pH 7.0 for all the detergents decreased as did that of EDTA, while the MIC of polymyxin increased 2-fold.

**Figure 7:**
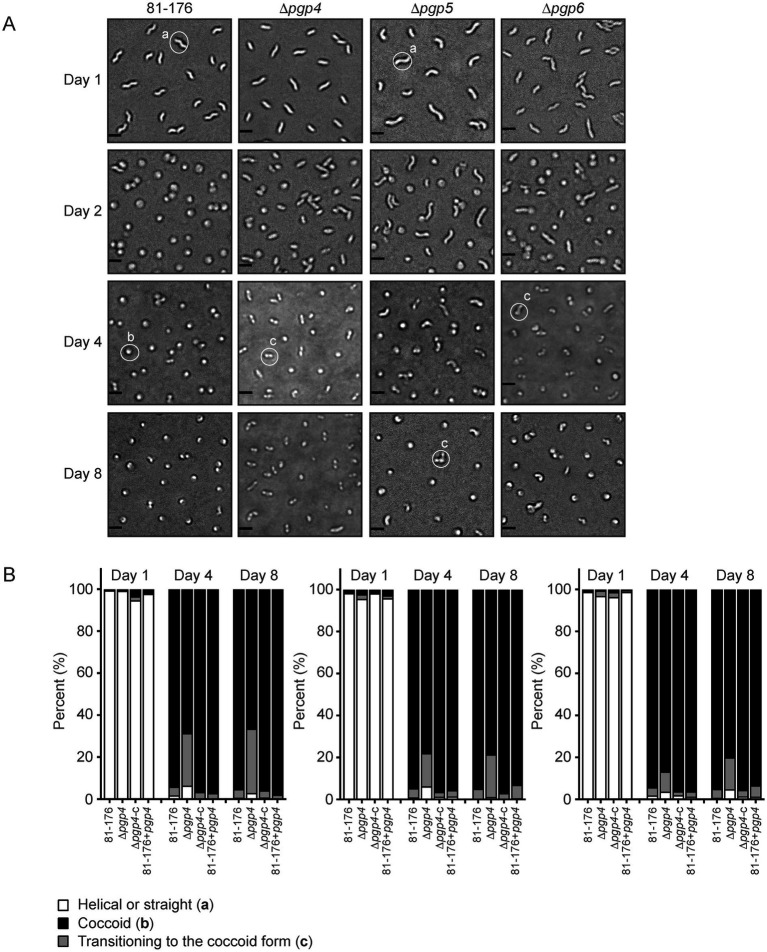
The effect of *pgp4, pgp5* and *pgp6* on the transition to the coccoid form. **(A)** DIC microscope images of *C. jejuni* wild type 81–176, ∆*pgp4*, ∆*pgp5* and *∆pgp6* mutant strains grown on solid media at 37°C to follow the transition to the coccoid form over time. Representative cells considered to be helical, coccoid or transitioning to the coccoid form are indicated by a, b or c, respectively. **(B)** The percentage of helical, coccoid and cells transitioning to the coccoid form as determined from DIC images such as those shown in **(A)** of the *pgp4*, *pgp5* and *pgp6* mutant, complemented (∆*pgp4c*, ∆*pgp5c*, and ∆*pgp6c*) and overexpression (81-176+*pgp4*, 81-176+*pgp5*, and 81-176+*pgp6*) strains. At least three separate fields of view of approximately 100–200 bacteria were counted for each strain at each timepoint and this was carried out in triplicate.

Coccoid formation during aging was monitored to compare the morphological transition between strains, as done previously (Frirdich et al., 2019; Frirdich et al., 2023). Cells were grown at 37°C on solid media under microaerophilic conditions and coccoid formation was examined at day 1, day 2, day 4 and day 8 by DIC microscopy (Figure 7A). Samples were taken from the center of the plate (sampling from different areas of the plate can show some variability). The percentage of helical, coccoid, and cells transitioning to the coccoid form was quantified from the DIC images (Figure 7B). The mutant strains were all impaired in the coccoid transition with a higher percentage of cells in the transitioning state and less in the coccoid form and a very slightly higher amount of cells still in the helical form. At day 8, 4.3% of wild type were in the transitioning state in contrast to 30.5% in ∆*pgp4*, 20.2% in ∆*pgp5* and 15.2% in ∆*pgp6*. The numbers of coccoid cells were 95.0% for wild type, 66.5% for ∆*pgp4*, 78.6% for ∆*pgp5* and 80.0% for ∆*pgp6* and the remaining helical cells were 0.6% for wild type, 3.0% for ∆*pgp4*, 1.2% for ∆*pgp5* and 4.8% for ∆*pgp6*. The complemented and overexpression strains were similar to wild type.

#### 3.2.2 The ∆*pgp5* strain, but not ∆*pgp4* nor ∆*pgp6*, shows a minor reduction in adherence, invasion and intracellular survival in epithelial cells

**Figure 8:**
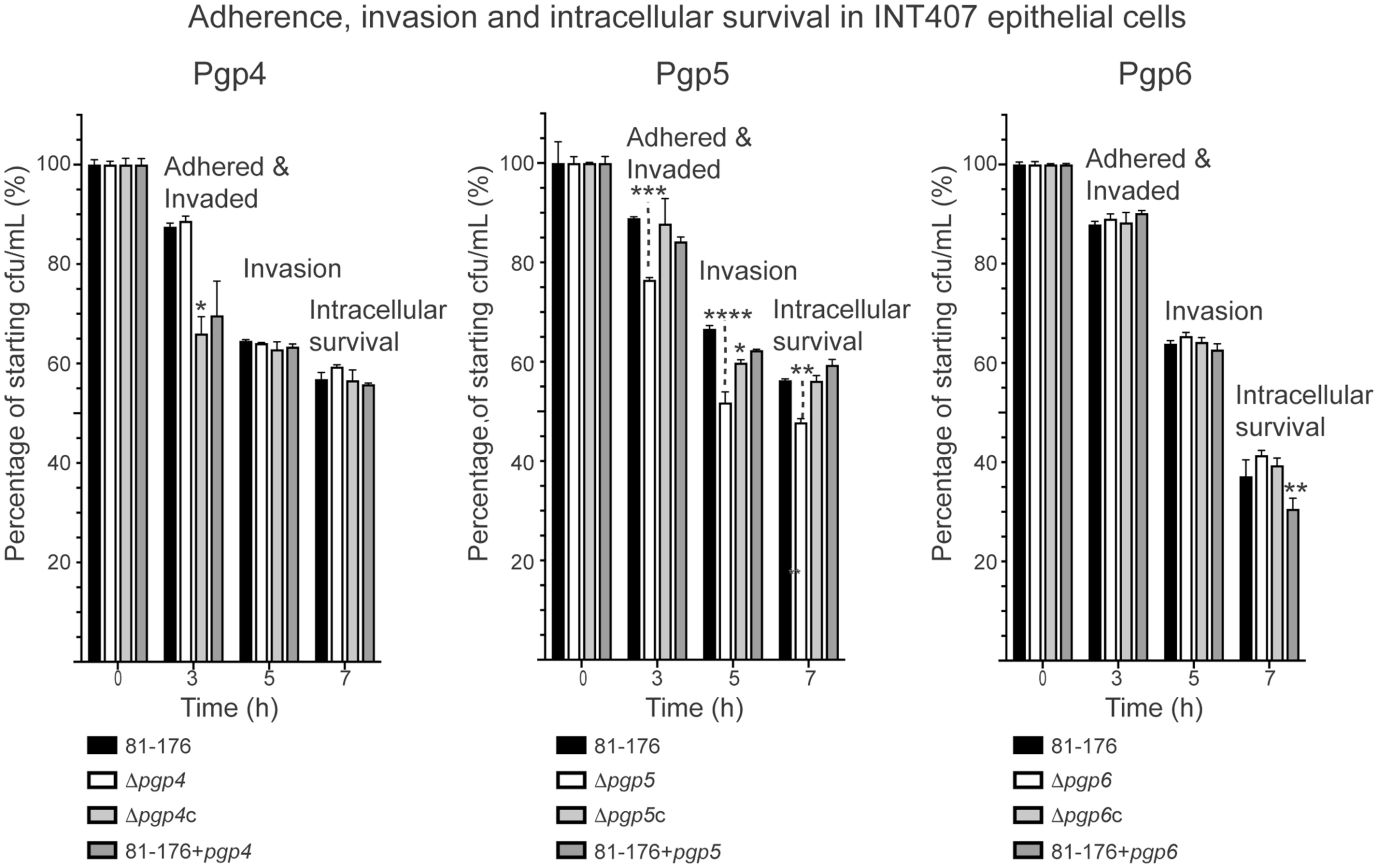
The effect of *pgp4, pgp5* and *pgp6* on adherence, invasion and intracellular survival in epithelial cells. The adherence, invasion and intracellular survival ability of the *pgp4*, *pgp5* and *pgp6* mutant, complemented (∆*pgp4c*, ∆*pgp5c*, and ∆*pgp6c*) and overexpression (81–176 + *pgp4*, 81–176 + *pgp5* and 81–176 + *pgp6*) strains in the INT407 epithelial cell line was assessed by a gentamicin (Gm) protection assay. Adherence and invasion was assessed at 3 h, invasion at 5 h (post-gentamicin treatment for 2 h), and intracellular survival at 7 h. The data was normalized with the cfu of the inoculum representing 100% and the cfu at each timepoint calculated as a percentage of the inoculum. In all experiments S.E. (error bars) were calculated from triplicate readings and are representative of three independent experiments. Statistical significance was calculated using a two-way ANOVA with the Dunnett’s test for multiple comparisons in comparison to wild type. The asterisk (*) indicates a statistically significant difference in comparison to wild type, with *, **, ***, or *** indicating *p* < 0.05, *p* < 0.01, *p* < 0.001 and *p* < 0.0001, respectively.

A gentamicin protection assay was used to assess the adherence, invasion, and intracellular survival properties of the *C. jejuni pgp4*, *pgp5* and *pgp6* mutant, complemented and overexpression strains in INT407 epithelial cells (Figure 8). Only the *pgp5* mutant showed a slight reduction in adherence, invasion and intracellular survival, as demonstrated previously (Stahl et al., 2016). The decrease in ∆*pgp5* adherence and invasion (t = 3 h) of 12.4% (in comparison to wild type) and intracellular survival (t = 7 h) of 8.5% was restored by complementation, while the reduction in invasion (t = 5 h) of 14.8% was partially restored by 8.0%. The adherence and invasion of ∆*pgp4*c and *pgp4* overexpression strains measured 3 h post-infection was 21.5 and 17.8% less than wild type, with only the difference between the complement and wild type being statistically significant. No differences at the subsequent invasion and intracellular survival time points were seen. The *pgp6* overexpression strain showed a statistically decrease of 17.6% in intracellular survival at 7 h post-infection in comparison to wild type.

## 4 Discussion

The Pgp4, Pgp5 and Pgp6 M23 peptidases have a role in determining the degree of curvature of the *C. jejuni* cell (Frirdich et al., 2023). Using PG cleavage assays, Pgp4 and Pgp5 were shown to have DD-EPase and DD-CPase activity, and Pgp6 to be a DD-CPase.

### 4.1 Comparison of *C. jejuni* Pgp4, Pgp5, and Pgp6 to *Helicobacter pylori* orthologs

Like *C. jejuni*, *Helicobacter pylori* is a member of the *ε*-Proteobacteria and has a helical morphology. Both organisms share some similar PG hydrolases, including Pgp1, Pgp2, Pgp4, Pgp5 and Pgp6. *H. pylori* homologs of Pgp1 (Csd4) and Pgp2 (Csd6) have similar enzymatic functions and roles in helical shape generation (Frirdich et al., 2012; Sycuro et al., 2012; Sycuro et al., 2013; Frirdich et al., 2014). *H. pylori* Csd1 and Csd3/HdpA are orthologs of *C. jejuni* Pgp4 and Pgp5, respectively. The Pgp6 ortholog in *H. pylori* has not been studied. From the muropeptide profile, Csd1 was shown to have DD-EPase activity (Sycuro et al., 2010) and no DD-CPase activity, unlike the *C. jejuni* ortholog. Biochemical activity assays may show that Csd1 indeed has DD-CPase activity. Like the *C. jejuni* ortholog, Csd3/HdpA has DD-EPase and DD-CPase activity that was confirmed biochemically (Bonis et al., 2010). Unlike with *C. jejuni*, deletion of the *H. pylori* Pgp4 and Pgp5 orthologs produced muropeptide profiles indicative of their activity (Bonis et al., 2010; Sycuro et al., 2010). This indicates that loss of Pgp4 or Pgp5 in *C. jejuni* likely affects other proteins in the PG biosynthetic complex which does not happen in *H. pylori*. Therefore, despite having similar enzymes and a similar helical morphology, the morphogenesis program of *H. pylori* and *C. jejuni* does likely differ.

### 4.2 *In vivo* effects of Pgp4, Pgp5, and Pgp6 on muropeptide structure

Taking into consideration the biochemical activity of Pgp4, Pgp5 and Pgp6, the corresponding mutant muropeptide profiles published previously (Frirdich et al., 2023) were reexamined as the muropeptide changes could not be explained at that time. However, none of the muropeptide changes could be attributed to the loss of DD-EPase and/or DD-CPase activity. Some muropeptide changes were contrary to what would be expected by deleting a DD-EPase cleaving cross-links. For example, in the ∆*pgp5* mutant, there was a decrease rather than an increase in cross-linking, increase in monomers and decrease in dimers and trimers. This would signify an increase in DD-EPase activity of another enzyme and highlights the complex interactions and regulatory pathways that are involved in PG biochemistry. The identification of protein-interaction partners is required to better understand the relationships between Pgp4, Pgp5 and Pgp6 and other PG biosynthetic enzymes and interpret the effect of deleting these enzymes on PG structure.

The *C. jejuni* muropeptide profile has very low levels of pentapeptides (Frirdich et al., 2012; Frirdich et al., 2014; Frirdich et al., 2019; Frirdich et al., 2023) meaning *C. jejuni* has high levels of DD-CPase activity. There are now four known *C. jejuni* PG hydrolases that have DD-CPase activity cleaving pentapeptides: Pgp3 (Min et al., 2020), Pgp4, and Pgp5 and Pgp6. Deletion of *pgp6* resulted in a muropeptide profile with increased pentapeptides with a total of 6.9% pentapeptides in contrast to 0.2–1.4% in the wild type, 0.8% in ∆*pgp4* and 0.7% in ∆*pgp5* (Frirdich et al., 2023). Following from this, of the three enzymes, Pgp6 is the primary enzyme responsible for cleaving the terminal D-Ala residue on the PG pentapeptide sidechain and neither Pgp4 or Pgp5 can compensate completely when Pgp6 is deleted. The muropeptide profile of ∆*pgp3* has yet to be determined so observations regarding Pgp3 DD-CPase activity in pentapeptide processing cannot be made. In contrast to *C. jejuni* with 0.2–1.4% pentapeptides, *H. pylori* has very low DD-CPase activity and very high levels of pentapeptides ranging from 46.4–62.9% depending on the strain and the preparation (Chaput et al., 2007; Sycuro et al., 2010; Sycuro et al., 2012; Chaput et al., 2016). Interestingly, the *H. pylori* Pgp6 ortholog has a degenerated M23 catalytic motif (HxxxD, GxH), where the first histidine residue of HxH is replaced with glycine. This is in contrast to the intact motifs found in *C. jejuni* and other bacteria. It is possible that the presence of an inactive *H. pylori* Pgp6 ortholog and its inability to process pentapeptides is an adaptation contributing to the high levels of pentapeptides in this organism.

### 4.3 Pgp6 model potentially explains novel DD-CPase activity

As far as we know, Pgp6 is the first M23 peptidase domain protein identified with DD-CPase and no DD-EPase activity. The AlphaFold model of Pgp6 reveals a potentially novel architecture within the M23 peptidase domain that would restrict access to the active site groove to smaller substrates (such as the monomeric pentapeptide) and exclude dimeric substrates. This would explain the lack of Pgp6 DD-EPase activity. This is in contrast to the DD-CPase and DD-EPase enzymes such as *C. jejuni* Pgp3 (Min et al., 2020) and *H. pylori* Csd3 (ortholog of Pgp5) (An et al., 2015) and the DD-EPase *H. pylori* Csd1 (ortholog of Pgp4) (An et al., 2016), and *V. cholerae* ShyA (Shin et al., 2020), which demonstrate open substrate access and can act on dimeric substrates (Figure 4). Structural studies on Pgp6 will confirm the AlphaFold model.

### 4.4 Role of Pgp4, Pgp5, and Pgp6 on *C. jejuni* physiology and pathogenesis attributes

Complete loss of helical cell shape in *C. jejuni* and the associated muropeptide changes have diverse effects on the physiology and pathogenesis of *C. jejuni* shown using rod-shaped ∆*pgp1* and ∆*pgp2* PG hydrolase mutants (Frirdich et al., 2012; Frirdich et al., 2014). Deletion of the *pgp4*, *pgp5* and *pgp6* genes results in strains that are still curved but with altered degrees of curvature (Frirdich et al., 2023). These mutants were used to examine the effects of changes in the amount of helical curvature on *C. jejuni* biology (summarized in Table 1). These changes were often distinct to those of ∆*pgp1* and ∆*pgp2*. The ∆*pgp5* mutant had the most drastic biological changes of the mutants (Table 1). It is also the only mutant with numerous changes in its PG muropeptide profile (Frirdich et al., 2023). These muropeptide changes were not explained by a lack of DD-EPase/DD-CPase activity, suggesting indirect effects of gene deletion on the PG composition. Reasons for this are suggested elsewhere (Frirdich et al., 2023). Note that for complementation and overexpression, the *pgp4*, *pgp5* and *pgp6* genes were inserted into the rRNA spacer locus and expression is driven from the Cm promoter from the pRRC plasmid and/or from the gene’s putative upstream promoter inserted with the gene. Therefore, gene expression and regulation will differ from that of the wild type. This may explain non-wild-type phenotypes sometimes seen with these strains.

**Table 1:** Summary of Pgp4, Pgp5, and Pgp6 enzyme function and *∆pgp4*, *∆pgp5* and *∆pgp6* mutant phenotypes.

Characteristics	*C. jejuni* PG hydrolase		
	Pgp4	Pgp5	Pgp6
Function	DD-EPase, DD-CPase	DD-EPase, DD-CPase	DD-CPase
Mutant morphology (Frirdich et al., 2023)	Decreased curvature (decreased angularity)	Variable curved rod morphologies with primarily C-and S-shape cells (increased angularity)	Pleomorphic morphology ranging from straight rods to slightly curved (decreased angularity)
Mutant muropeptide profile (Frirdich et al., 2023)	Decrease in Tri-	Extensive changes: Decreased Di- Increased Tri-, Tetra- Increased monomers Decreased dimers and trimers Decrease in anhydro chain ends resulting in an increase in glycan stand length Decreased cross-linking	Increase in Penta-, TetraPenta- (unique) Decrease in Di- Increase in Tri- Decrease in trimers Decrease in anhydro chain ends resulting in an increase in glycan stand length
Biological properties of the mutant (in comparison to wild type)			
Motility	No change	14.9% decrease	7.9% decrease
Biofilm formation (at day 3)	2.7-fold lower	1.5-fold higher	No change at day 3, but 1.4-fold lower at day 2
CFW reactivity	Hypofluorescent	No change	No change
Autoagglutination	Faster rate of autoagglutination with an increase in autoagglutination of 8.0% by 24 h.	Slower rate of autoagglutination with increase in autoagglutination of 2.2% by 24 h.	Faster rate of autoagglutination with increase in autoagglutination of 7.6% by 24 h.
Hydrophobicity	No change	No change	No change
Transition to coccoid (at day 8)	More cells in the transitioning state: 30.5% in comparison to 4.3% in wild type.	More cells in the transitioning state: 20.2% in comparison to 4.3% in wild type.	More cells in the transitioning state: 15.2% in comparison to 4.3% in wild type.
pH survival	No change	No change (Note: increased Pgp5 levels show slight decrease in acid survival at pH 5.0)	No change (Note: increased Pgp6 levels show slight decrease in acid survival at pH 5.0)
MIC to detergents, antimicrobial compounds, chelating agents and salts	No change	No change	No change
Host interaction phenotypes of the mutant (in comparison to wild type)			
Adherence, invasion and intracellular survival in epithelial cells	No change	Minor reduction. Adherence and invasion at t = 3 reduced by 12.4%, invasion at t = 5 by 14.8% and invasion and intracellular survival at t = 7 by 8.5%	No change
IL-8 release (Stahl et al., 2016)		2.6-fold decrease at 24 h	
Crypt colonization in SIGIRR−/−mouse model (Stahl et al., 2016)		No change	

Extensive analysis of the biological properties of a mutant lacking the fourth *C. jejuni* M23 peptidase Pgp3 have yet to be carried out, with only the morphology and intracellular survival of the mutant having been examined (Min et al., 2020). Loss of the Pgp3 M23 peptidase resulted in a curved rod morphology (Min et al., 2020) with a decrease in angularity that wasn’t as pronounced as that of ∆*pgp4* and ∆*pgp6* (J. Vermeulen, T. Ibitsam and E. Frirdich, unpublished results) and a marked defect in invasion and intracellular survival in Caco2 epithelial cells (after 3 h infection followed by 3 h gentamicin treatment to kill extracellular bacteria) in comparison to wild type (Min et al., 2020). However, in their gentamicin protection assay Min et al. (2020) lysed the wells containing cells infected with *C. jejuni* with 0.1% Triton X-100 as opposed to water run through a 25-gauge syringe (a protocol developed for *C. jejuni* by E.C. Gaynor as Triton X-100 was found to affect the viability of some *C. jejuni* wild type strains with some mutants being even more sensitive to Triton). The use of Triton may affect the results of Min et al. (2020) and the validity of the ∆*pgp3* defect in invasion and intracellular survival.

#### 4.4.1 Motility

*C. jejuni* motility is a key virulence determinant in host colonization facilitating penetration of the highly viscous mucosal layer of the gastrointestinal tract (Tikhomirova et al., 2024). Motility is also required for chemotaxis. Chemotaxis is important for *C. jejuni* host invasion and environmental survival (Korolik, 2019). In soft agar, the motility of rod shaped ∆*pgp1* and ∆*pgp2* mutants was 82.5% and 73.7% of wild type, respectively. In the curved rod mutants, the motility of ∆*pgp4* was similar to wild type and that of ∆*pgp6* 92.1% of wild type, while that of ∆*pgp5* was more pronounced being 85.1% of wild type and more similar to that of ∆*pgp1*. The morphology of the ∆*pgp5* mutant had an increase in angularity (or mean curvature), while the ∆*pgp4* and ∆*pgp6* had decreased angularity, potentially contributing to the variation in motility defects (see Supplementary Figure 1 for morphology) (Frirdich et al., 2023). *C. jejuni* has amphitrichous flagella or a flagellum at each cell pole. A recent study on how *C. jejuni* coordinates the rotation of each opposing flagellum during movement has identified an unappreciated role for the *C. jejuni* helical shape in *C. jejuni* flagellar motility (Cohen et al., 2020). In high viscosity media, *C. jejuni* wraps the leading left handed flagellar filament around the right handed helix of the cell body to increase swimming speeds (Cohen et al., 2020). Directional changes involve unwrapping of the filament from the cell body with the helical shape of opposite handedness to the filament being required for efficient unwrapping, as the rod shaped ∆*pgp1* mutant was defective for flagellar unwrapping (Cohen et al., 2020). Flagella unwrapping ability of the curved rod mutants with different helical shapes may be altered and could affect motility.

#### 4.4.2 Biofilm formation

Biofilm formation ensures *C. jejuni* transmission and persistence in the environment and in the human gastrointestinal tract (Tikhomirova et al., 2024). Motility does have a role in biofilm formation in *C. jejuni* (Puning et al., 2021; Ma et al., 2022). The rod-shaped ∆*pgp1* and ∆*pgp2* mutants with reduced motility also displayed reduced biofilm formation (Frirdich et al., 2012; Frirdich et al., 2014). This correlation was restricted to the rod-shaped mutants. A mutant in the O-acetylpeptidoglycan esterase *ape1* with altered cell curvature had a slight motility defect but was a hyper-biofilm former (Ha et al., 2016). Similarly, ∆*pgp5*, with a more similar morphology to ∆*ape1* than ∆*pgp4* and ∆*pgp6*, also had a motility defect and produced higher biofilm levels. Despite the lack of a motility defect, biofilm formation was reduced in ∆*pgp4* to greater levels than ∆*pgp1* and ∆*pgp2*. There was no change in ∆*pgp6* biofilm formation even though this mutant had slightly reduced motility. Surface hydrophobicity is also a critical factor influencing biofilm formation in *C. jejuni* (Ma et al., 2022). This was unchanged in ∆*pgp4,* ∆*pgp5* and ∆*pgp6* (as well as ∆*pgp1* and ∆*pgp2*). These results indicate that biofilm formation with the curved rod mutants was affected by a factor other than motility and cell surface hydrophobicity.

#### 4.4.3 CFW fluorescence

CFW is a fluorescent dye binding *β* (1,3) and β (1,4) glycosidic bonds on the cell surface. We had previously identified CFW hypo- and hyper-reactive (*dim* and *brt*) mutants with changes in cell surface attributes and defective for various aspects of pathogenesis such as biofilm formation (McLennan et al., 2008; Naito et al., 2010; Frirdich et al., 2012; Frirdich et al., 2014). A correlation was established between CFW reactivity and biofilm formation with CFW hyperfluorescent strains being hyperbiofilm forming and conversely, CFW hypofluorescent strains (such as ∆*pgp1* and ∆*pgp2*) being hypobiofilm forming (McLennan et al., 2008; Naito et al., 2010; Frirdich et al., 2012; Frirdich et al., 2014). This held true for the ∆*pgp4* mutant that had reduced biofilm formation and was hypofluorescent on CFW, but not ∆*pgp5* that produced increased biofilms but no change in CFW reactivity. Biofilms produced by the ∆*pgp6* mutant were not significantly different to wild type at day 3 and showed wild type CFW reactivity. The exact *C. jejuni* polymer recognized by CFW in *C. jejuni* CFW fluorescence assays is unknown, but CFW does recognize the β(1–4) linkages of the PG backbone (Frirdich et al., 2017). Our current hypothesis is that CFW accessibility to the PG backbone is what dictates CFW fluorescence (Frirdich et al., 2017). Passage on CFW was found to select for mutations in *pgp1*, *pgp2* and *pgp4* that are all hypofluorescent on CFW, thereby being more resistant to the stress caused by CFW (Frirdich et al., 2017).

#### 4.4.4 Autoagglutination

*C. jejuni* autoagglutination is associated with full-length, glycosylated flagella, but not motility directly (Misawa and Blaser, 2000; Golden and Acheson, 2002; Guerry et al., 2006; Ewing et al., 2009). The structure of the *C. jejuni* flagellar motor is also important for mediating flagellar disentanglement which affects agglutination levels (Cohen et al., 2024). By electron microscopy, the ∆*pgp4*, ∆*pgp5*, and ∆*pgp6* mutants did not display any obvious changes in flagellar structure or number (Frirdich et al., 2023). However, more subtle flagellar changes such as in the flagellar motor or changes in flagellar glycosylation could explain the differences in agglutination. Also, the *C. jejuni* helical shape minimizes interactions between the cell body and flagellar filament that contribute to autoagglutination (Cohen et al., 2020). The changes to the helical shape in the ∆*pgp4*, ∆*pgp5*, and ∆*pgp6* mutants could thereby affect the autoagglutination ability of these strains. The ∆*pgp4* and ∆*pgp6* mutants which have a curved morphology with decreased angularity in comparison to wild type (Frirdich et al., 2023) agglutinated faster than wild type. The ∆*pgp5* mutant with morphology with increased angularity in comparison to wild type (Frirdich et al., 2023) displayed altered autoagglutination kinetics agglutinating more slowly than wild type but then agglutinating to slightly higher levels than wild type by 24 h. It is possible that the particular morphology of ∆*pgp5* with an increased angularity affects its autoagglutination kinetics. The *pgp4* overexpression strain also showed decreased autoagglutination, but only at the 6 h time point. Like ∆*pgp5,* this strain had increased angularity, but an overall different morphology to that of ∆*pgp5* (Frirdich et al., 2023). All the complemented strains and overexpression strains had altered autoagglutination kinetics at certain timepoints. While they did not have changes in angularity to explain the autoagglutination results, they may have slightly altered morphologies due to PG hydrolase expression differences that could affect agglutination. *C. jejuni* autoagglutination has been correlated with bacterial hydrophobicity and adherence to INT407 cells (Misawa and Blaser, 2000; Golden and Acheson, 2002; Guerry et al., 2006). Cell surface hydrophobicity was unaffected in all the mutants. Decreased adherence to INT407 cells was seen with the ∆*pgp5* mutant, which did have decreased agglutination at 3 h.

#### 4.4.5 Transition to coccoid form

*C. jejuni* transitions to a coccoid form as it ages and under environmental stresses such as starvation, temperature, pH, and osmolarity (Svensson et al., 2008; Jackson et al., 2009; Ikeda and Karlyshev, 2012; Frirdich and Gaynor, 2013; Frirdich et al., 2019). The exact role of *C. jejuni* coccoid formation in environmental survival and pathogenesis is still unclear. However, *C. jejuni* do become coccoid within epithelial cells and coccoid *C. jejuni* are non-motile and non-infectious (Frirdich et al., 2019). Previously, the DL-CPase Pgp1 and the amidase AmiA were shown to be key for coccoid formation, but not the LD-CPase Pgp2 (Frirdich et al., 2019). The ∆*pgp1*, ∆*amiA* and ∆*pgp1*∆*amiA* mutants had higher levels of helical cells after 8 days of culture in comparison to wild type and were defective in initiation of the coccoid form (Frirdich et al., 2019). In contrast, the ∆*pgp4*, ∆*pgp5* and ∆*pgp6* were inhibited in completion of the transitioning state to the coccoid form with an accumulation of transitioning cells in comparison to wild type after 8 days of growth. Coccoid PG showed increased levels of dipeptides and decreased tri- and tetrapeptides with no change in the degree of cross-linking and the level of monomers, dimers and trimers (Frirdich et al., 2019). These changes are not a direct result of the DD-CPase and/or DD-EPase activity of Pgp4, Pgp5 and Pgp6.

#### 4.4.6 Acid survival

Acid survival is an important factor affecting the ability of an enteric pathogen to survive passage through the gastrointestinal tract. As stated previously, some PG hydrolases are important for growth in acidic pH (Egan et al., 2020). Loss of *pgp4*, *pgp5* and *pgp6*, as well as *pgp1* and *pgp2* (data not shown), did not affect survival at low pH. This is supported by previous studies. In *C. jejuni* strain 11168, expression of *pgp4*, *pgp5* or *pgp6* were unchanged in response to acid shock (Reid et al., 2008a) or during growth under mildly acidic conditions (pH 5.5, 6.0 and 6.5) (Reid et al., 2008b). Also, no transposon mutants in *pgp4*, *pgp5* or *pgp6* were identified as having impaired growth at pH 5.5 or pH 6.0 (Reid et al., 2008b). However, a proteomics study using *C. jejuni* 11168 showed the reduced abundance of the Pgp6 protein when *C. jejuni* was grown under mildly acidic conditions (pH 5.8) and subjected to acid shock (pH 4.0, 2 h) (Ramires et al., 2023). This correlates with our results that higher levels of Pgp6 in the *pgp6* overexpression strain were slightly detrimental to acid survival at pH 5.0. The same was seen with Pgp5 with the complemented and overexpression strain both having slightly reduced growth at pH 5.0. The levels of Pgp5 in the complemented strain could be higher than those in the wild type possibly explaining the defect in this strain. How Pgp5 and Pgp6 can adversely affect survival at acidic pH remains to be established.

#### 4.4.7 Outer membrane stability

Similar to *∆pgp1* and ∆*pgp2*, the ∆*pgp4, ∆*pgp5 and ∆*pgp6* mutants were not more sensitive to compounds that would indicate differences in outer membrane stability in comparison to wild type. Changes in *C. jejuni* outer membrane permeability occurred when the pH was dropped from 7.0 to 5.0, signified by increased susceptibility to the chelating agent EDTA and detergents. At pH 5.0, polymyxin B resistance increased two-fold. Polymyxin is a cationic peptide that binds to the negatively charged lipopolysaccharide. Increased resistance to polymyxin B at pH 5.0 could indicate a change in the charge or fluidity of the outer membrane (Trimble et al., 2016). Interestingly, all strains including wild type were more sensitive to MES at pH 5.0 in comparison to pH 7.0 with an MIC_50_ of 50 mM at pH 5.0. MES at a concentration of 100 mM was often used to study *C. jejuni* responses to acidic pH and acid survival (Reid et al., 2008a; Reid et al., 2008b) due to its buffering capacity at pH 5.5–7.0. These results may be confounded by the negative affects of MES at low pH.

#### 4.4.8 Adherence, invasion and intracellular survival in epithelial cells

Only the ∆*pgp5* mutant had a decrease albeit minor in adherence, invasion and intracellular survival in cultured epithelial cells replicating our previous results (although with complementation to wild type levels in this work) (Stahl et al., 2016). This mutant also had the most significant motility defect and impaired autoagglutination, factors important in targeting and adherence to host cells, respectively. In a previous study, ∆*pgp5* PG showed decreased secretion of the proinflammatory chemokine IL-8 from epithelial cells (Stahl et al., 2016). In that study, the ∆*pgp5* mutant along with the rod-shaped ∆*pgp1* and ∆*pgp2* mutants were used to examine the role of helical shape in colonization ability and inflammation in a *Sigirr*−/− mouse model of *C. jejuni* intestinal infection (described in the Introduction) (Stahl et al., 2016). The ∆*pgp5* mutant was selected as the representative curved rod mutant as it had the most drastic and consistent changes to the curvature within the bacterial population (Stahl et al., 2016). In the *Sigirr*−/− mouse model, despite the impaired host *in vitro* phenotypes, the ∆*pgp5* mutant could penetrate the mucus overlaying the intestinal epithelium and crypts, colonize and trigger an inflammatory response similar to wild type and unlike ∆*pgp1* and ∆*pgp2* that were non-pathogenic (Stahl et al., 2016). Interestingly, ∆*pgp1* and ∆*pgp2* were not defective for adherence, invasion and intracellular survival in epithelial cells (Frirdich et al., 2012; Frirdich et al., 2014). As shown in this study, neither were the *pgp4* nor the *pgp6* mutants. The ∆*pgp4c* complemented strain had reduced adherence and invasion (at the 3 h time point), but then showed wild type invasion at 5 h and intracellular survival at 7 h. This is a confounding observation as decreased adherence would be expected to accompany a decrease in invasion and intracellular survival but these were similar to wild type. One possible explanation is that the decreased adherence is a result of the decreased motility of this strain (Figure 5A). The ∆*pgp4c* complemented strain may have an increased ability to invade and survive intracellularly in comparison to wild type that makes up for the adherence defect and then results in wild type invasion and intracellular survival at later timepoints. The *pgp6* overexpression strain was slightly reduced in intracellular survival at 7 h post-infection. Interestingly, this strain was also sensitive to low pH. However, *C. jejuni* survives intracellularly by avoiding delivery to the acidic lysosomes (Watson and Galan, 2008), so the importance of pH resistance in *C. jejuni* intracellular survival is unknown.

Another group identified the *pgp4* (*1105*) gene as having a role in *C. jejuni* cell curvature in a transposon mutant library visual screen for cell shape mutants (Esson et al., 2017). They found that the mutant had a curved rod phenotype visually similar to that of our ∆*pgp4* mutant, although morphological analyses were not carried out. Their ∆*pgp4* mutant in the same 81–176 wild type strain as the one used in our experiments had a motility defect and reduced adherence and invasion *in vitro* in cultured Caco2 cells (Esson et al., 2017), while our ∆*pgp4* mutant had neither a motility nor an adherence or invasion defect in INT407 cells. The cause of phenotypic differences between our two ∆*pgp4* mutant strains is unclear. Esson et al. (2017) showed that their ∆*pgp4* had wild type chick colonization.

## 5 Conclusion

This study expands on previous work showing the significance of helical shape in *C. jejuni* pathogenesis to include the importance of the degree of cell curvature. Each curved mutant showed distinct phenotypic changes (Table 1), indicating they are not functionally redundant and each play a distinct role in *C. jejuni* helical shape determination and pathogenesis. Subsequent studies identifying interaction partners for *Pgp1*–6 as well as protein localization studies will be key in determining how these proteins are coordinated to generate the *C. jejuni* helical shape.

## Data Availability

The original contributions presented in the study are included in the article/Supplementary material, further inquiries can be directed to the corresponding author.

## Footnotes

^1^ https://cmdr.ubc.ca/bobh/method/modified-mic-method-for-cationic-antimicrobial-peptides/

^2^ https://blast.ncbi.nlm.nih.gov/Blast.cgi
