# Supplementary material for: Novel M23 peptidases Pgp4, Pgp5, and Pgp6 contribute to helical cell shape in Campylobacter jejuni
Source: Front Microbiol. 2025 Sep 9;16:1641976. doi: 10.3389/fmicb.2025.1641976 (PMC12454360; doi:10.3389/fmicb.2025.1641976)
Supplement: Supplementary file 1 [file Data_Sheet_1.docx]

***Supplementary Material***

**Three novel M23 peptidases contribute to helical cell shape and pathogenesis in *C. jejuni***

**Chang Sheng-Huei Lin^1†^, Jenny Vermeulen^1†^, Jacob Biboy^2^, Erin C. Gaynor^1§^, Waldemar Vollmer^2,3^, and Emilisa Frirdich^1*^**

† These authors share first authorship

^§^ Deceased March 6, 2023

*** Correspondence:**

Emilisa Frirdich

[emilisa.frirdich@mail.ubc.ca](mailto:emilisa.frirdich@mail.ubc.ca)

**List of Contents**

**1. Supplemental Tables**

***Supplementary Table 1****.* Bacterial strains and plasmids.

***Supplementary Table 2****.* Primers.

***Supplementary Table 3****.* Pgp6 orthologs used in this study

***Supplementary Table 4****.* MIC_50_ as determined by microtiter broth dilution method

**2. Supplemental Figures**

***Supplementary Figure 1****.* Morphology of *C. jejuni* *pgp4*, *pgp5*, and *pgp6* mutant, complement and overexpressor strains

***Supplementary Figure 2****.* Topology of the Pgp6 M23 Peptidase Domain

***Supplementary Figure 3****.* Confidence of the Pgp6 AlphaFold Model (A0A0H3PIR6)

**3. References**

**1. Supplemental Tables**

**Supplementary Table 1.** Bacterial strains and plasmids

| **Strain or Plasmid** | **Genotype, serotype or description** | **Reference or Source** |
| --- | --- | --- |
| ***C. jejuni*** |  |  |
| 81-176 | Wild type isolated from a diarrheic patient | (Korlath et al., 1985) |
| Δ*pgp4 (1105*) | 81-176 *1105*::*aphA3*; Km^R^ | (Frirdich et al., 2017) |
| Δ*pgp5 (1228*) | 81-176 *1228*::*aphA3*; Km^R^ | (Stahl et al., 2016) |
| Δ*pgp6 (0166*) | 81-176 *0166*::*aphA3*; Km^R^ | (Frirdich et al., 2023) |
| Δ*pgp4c* | 81-176 *1105*::*aphA3 rrn::1105;* Km^R^ Cm^R^ | (Frirdich et al., 2017) |
| Δ*pgp5c* | 81-176 *1228*::*aphA3 rrn::1228*; Km^R^ Cm^R^ | (Stahl et al., 2016) |
| Δ*pgp6c* | 81-176 *0166*::*aphA3 rrn::0166*; Km^R^ Cm^R^ | (Frirdich et al., 2023) |
| 81-176+*pgp4* | 81-176 *rrn::1105*; Cm^R^ | (Frirdich et al., 2023) |
| 81-176+*pgp5* | 81-176 *rrn::1228*; Cm^R^ | (Frirdich et al., 2023) |
| 81-176+*pgp6* | 81-176 *rrn::0166*; Cm^R^ | (Frirdich et al., 2023) |
| ***E. coli*** |  |  |
| DH5α | F^-^, φ80d *deoR lacZ*Δ*M15 endA1 recA1 hsdR17*(r_K_-m_K_+) *supE44 thi-1 gyrA96 relA1* Δ*(lacZYA-argF) U169* | Invitrogen |
| C41(DE3) | F-, *ompT hsdS*_B_(r_B_^-^ m_B_^-^) *gal dcm* (DE3) | Sigma |
| BL21(DE3)pLysS | F^-^ *ompT hsdS*_B_(r_B_^-^ m_B_^-^) *gal dcm* (DE3) pLysS; Cm^R^ | Novagen |
| Rosetta2(DE3)pLysS | F-, *ompT hsdS*_B_(r_B_^-^ m_B_^-^) *gal dcm* (DE3) pLysSRARE2; Cm^R^ | Novagen |
| **Plasmids** |  |  |
| pET28a | Bacterial expression vector; Km^R^ | Novagen |
| pET28a-*pgp4* | pET28a-1105 (aa 52-300) with a C-terminal His6 tag; Km^R^ | This study |
| pET28a-*pgp5* | pET28a-1228 (aa 20-379) with an N-terminal His6 tag; Km^R^ | This study |
| pET28a-*pgp6* | pET28a-0166 (aa 33-457) with a C-terminal His6 tag; Km^R^ | This study |

**Supplementary Table 2.** Primers

| **Primer** | **Sequence 5′ to 3′**  **(enzyme cutting sites are highlighted in bold)** | **Restriction Site** | **Reference** |
| --- | --- | --- | --- |
| 1105-6 | gaagaa**TCATGA**GCAAACTTAGCGATATCAGTG | *BspHI* | This study |
| 1105-2 | gaagaa**CTCGAG**CTGTTGTTTCTGAGCTAGAGCTG | *XhoI* | This study |
| optimized 0166-a | gaagaA**CCATGG**GCAAAAACCCGCCG | *NcoI* | This study |
| optimized 0166-b | gaagaa**CTCGAG**GTTTTTGCCCAGAATAATTTTTTTGCC | *XhoI* | This study |
| optimized 1228-a | ggaattc**CATATG**GTGGAAGAACTGACCTGGGATAACGG | *NdeI* | This study |
| optimized 1228-d | gaagaa**CTCGAG**TTAATCTTCTTCTTTCGGCGGGTTCGG | *XhoI* | This study |

**Supplementary Table 3**. Pgp6 orthologs used in this study

| **New phylum^1^** | **Old classification** | **Species** | **NCBI Protein Locus** | **Coverage** | **Identity** | **E-value** |
| --- | --- | --- | --- | --- | --- | --- |
| C | ε | C. jejuni 81-176 | CJJ81176_0166  (Pgp6) | - | - | - |
| C | ε | Helicobacter pylori 26695 | C694_05450 | 97% | 27% | 1e-43 |
| C | ε | Wolinella succinogenes DSM1740 | WS2011 | 98% | 40% | 2e-116 |
| C | ε | Arcobacter butzleri RM4018 | Abu_2038 | 98% | 33% | 4e-84 |
| C | ε | Sulfurimonas autotrophica DSM 16294 | Saut_0697 | 99% | 44% | 1e-139 |
| C | ε | Sulfurospirillum deleyianum DSM 6946 | Sdel_0242 | 98% | 46% | 1e-145 |
| C | ε | Sulfuricurvum kujiense DSM 16994 | Sulku_0455 | 98% | 44% | 1e-135 |
| C | ε | Sulfurovum sp. NBC37-1 | SUN_1977 | 99% | 41% | 2e-116 |
| C | ε | Nitratiruptor sp. SB155-2 | NIS_0417 | 98% | 46% | 5e-151 |
| C | ε | Nautilia profundicola AmH | NAMH_1549 | 94% | 42% | 1e-108 |
| C | δ^2^ | Hippea maritima DSM 10411 | Hipma_0325 | 97% | 30% | 1e-59 |
| C | δ^2^ | Desulfurella acetivorans A63 | DESACE_01445 | 97% | 28% | 7e-52 |
| T | δ | Desulfovibrio desulfuricans ND132 | DND132_0252 | 92% | 32% | 7e-64 |

^1^ The names of phylum are based on those of Oren and Garrity (Oren and Garrity, 2021). The letters “*C*“ and “*T*” represent *Campylobacterota* and *Thermodesulfobacteriota*.

*^2^* A phylogenetic study using 16s rRNA, 23s rRNA, and protein markers classify Hippea maritima and Desulfurella acetivorans into the Campylobacterota (Waite et al., 2017).

**Supplementary Table 4.** MIC_50_ as determined by microtiter broth dilution method.

|  | **MIC_50_** | | | | | | | | | | |
| --- | --- | --- | --- | --- | --- | --- | --- | --- | --- | --- | --- |
|  | **81-176** | | **∆0166** | | | | **∆1105** | | | **∆1228** | |
| **Compound** | **pH 5.0** | **pH 7.0** | | **pH 5.0** | **pH 7.0** | **pH 5.0** | | **pH 7.0** | **pH 5.0** | | **pH 7.0** |
| **Detergents** |  |  | |  |  |  | |  |  | |  |
| SDS (%) | 0.00625 | 0.1 | | 0.00625 | 0.1 | 0.00625 | | 0.1 | 0.00625 | | 0.05 |
| Triton X-100 (%) | 2 | >4 | | 1 | >4 | 1 | | >4 | 1 | | >4 |
| Tween20 (%) | 0.25 | 4 | | 0.5 | 4 | 0.5 | | 4 | 0.125-0.25 | | 4 |
| Porcine bile extract (mg/mL) | 20 | >20 | | 20 | >20 | 20 | | >20 | 20 | | >20 |
| MES (mM) | 50 | 200- >200 | | 50 | 200 | 50 | | 200 | 25 | | 200 |
| **Antimicrobials** |  |  | |  |  |  | |  |  | |  |
| Ampicillin (mg/mL) | 0.1 | 0.2 | | 0.1 | 0.1 | 0.1 | | 0.1 | 0.1 | | 0.1 |
| Polymyxin B (mg/mL) | 0.5 | 0.25 | | 0.5 | 0.25 | 0.5 | | 0.25 | 0.5 | | 0.25 |
| Protamine (mg/mL) | 0.5 | 0.5 | | 0.5 | 0.5 | 0.5 | | 0.5 | 0.5 | | 0.5 |
| **Chelating agent** |  |  | |  |  |  | |  |  | |  |
| EDTA (mM) | 0.0195 | 0.156-0.313 | | 0.0195 | 0.156 | 0.0195 | | 0.156 | 0.0098 | | 0.156 |
| **Salts** |  |  | |  |  |  | |  |  | |  |
| NaCl (M) | 0.25 | 0.125-0.25 | | 0.25 | 0.125-0.25 | 0.25 | | 0.25 | 0.25 | | 0.25 |
| MgCl_2_ (M) | 0.25 | 0.25 | | 0.25 | 0.25 | 0.25 | | 0.25 | 0.25 | | 0.25 |

**2. Supplemental Figures**

**Supplementary Figure 1. Morphology of *C. jejuni* *pgp4*, *pgp5*, and *pgp6* mutant, complement and overexpression strains.** DIC microscope images of the helical *C. jejuni* 81-176 strain, the ∆*pgp4*, ∆*pgp5*, and ∆*pgp6* mutant strains and corresponding complemented (∆*pgp4c*, ∆*pgp5c*, and ∆*pgp6c*) and overexpression strains (denoted +*pgp4*, +*pgp5*, and +*pgp6*). The scale bar indicates 2 μm.

**Supplementary Figure 2. Topology of the Pgp6 M23 Peptidase Domain**. Protein topology diagram illustrating the linker region and M23 peptidase domain in the Pgp6 AlphaFold model. This diagram was generated using PDBsum based on the Pgp6 AlphaFold model, with the final assignment of secondary structures manually verified in PyMOL.

**Supplementary Figure 3. Confidence of the Pgp6 AlphaFold Model (A0A0H3PIR6).** (A) ribbon representation of Pgp6, colored according to the pLDDT score. (B) predicted aligned error (PAE) obtained from the AlphaFold website.

**3. References**

Frirdich, E., Biboy, J., Huynh, S., Parker, C.T., Vollmer, W., and Gaynor, E.C. (2017). Morphology heterogeneity within a *Campylobacter jejuni* helical population: the use of calcofluor white to generate rod-shaped *C. jejuni* 81-176 clones and the genetic determinants responsible for differences in morphology within 11168 strains. *Mol Microbiol* 104(6)**,** 948-971. doi: 10.1111/mmi.13672.

Frirdich, E., Vermeulen, J., Biboy, J., Vollmer, W., and Gaynor, E.C. (2023). Multiple *Campylobacter jejuni* proteins affecting the peptidoglycan structure and the degree of helical cell curvature. *Front Microbiol* 14**,** 1162806. doi: 10.3389/fmicb.2023.1162806.

Korlath, J.A., Osterholm, M.T., Judy, L.A., Forfang, J.C., and Robinson, R.A. (1985). A point-source outbreak of campylobacteriosis associated with consumption of raw milk. *J Infect Dis* 152(3)**,** 592-596.

Oren, A., and Garrity, G.M. (2021). Valid publication of the names of forty-two phyla of prokaryotes. *Int J Syst Evol Microbiol* 71(10). doi: 10.1099/ijsem.0.005056.

Stahl, M., Frirdich, E., Vermeulen, J., Badayeva, Y., Li, X., Vallance, B.A., et al. (2016). The Helical Shape of *Campylobacter jejuni* Promotes *In Vivo* Pathogenesis by Aiding Transit through Intestinal Mucus and Colonization of Crypts. *Infect Immun* 84(12)**,** 3399-3407. doi: 10.1128/IAI.00751-16.

Waite, D.W., Vanwonterghem, I., Rinke, C., Parks, D.H., Zhang, Y., Takai, K., et al. (2017). Comparative Genomic Analysis of the Class Epsilonproteobacteria and Proposed Reclassification to Epsilonbacteraeota (phyl. nov.). *Front Microbiol* 8**,** 682. doi: 10.3389/fmicb.2017.00682.
